# Nasal alum-adjuvanted vaccine promotes IL-33 release from alveolar epithelial cells that elicits IgA production via type 2 immune responses

**DOI:** 10.1371/journal.ppat.1009890

**Published:** 2021-08-30

**Authors:** Eita Sasaki, Hideki Asanuma, Haruka Momose, Keiko Furuhata, Takuo Mizukami, Isao Hamaguchi

**Affiliations:** 1 Department of Safety Research on Blood and Biological Products, National Institute of Infectious Diseases, Musashi–Murayama, Tokyo, Japan; 2 Center for Influenza and Respiratory Virus Research, National Institute of Infectious Diseases, Musashi–Murayama, Tokyo, Japan; University of North Carolina at Chapel Hill, UNITED STATES

## Abstract

Aluminum hydroxide salts (alum) have been added to inactivated vaccines as safe and effective adjuvants to increase the effectiveness of vaccination. However, the exact cell types and immunological factors that initiate mucosal immune responses to alum adjuvants are unclear. In this study, the mechanism of action of alum adjuvant in nasal vaccination was investigated. Alum has been shown to act as a powerful and unique adjuvant when added to a nasal influenza split vaccine in mice. Alum is cytotoxic in the alveoli and stimulates the release of damage-associated molecular patterns, such as dsDNA, interleukin (IL)-1α, and IL-33. We found that Ag-specific IgA antibody (Ab) production was markedly reduced in IL-33-deficient mice. However, no decrease was observed in Ag-specific IgA Ab production with DNase I treatment, and no decrease was observed in IL-1α/β or IL-6 production in IL-33-deficient mice. From the experimental results of primary cultured cells and immunofluorescence staining, although IL-1α was secreted by alveolar macrophage necroptosis, IL-33 release was observed in alveolar epithelial cell necroptosis but not in alveolar macrophages. Alum- or IL-33-dependent Ag uptake enhancement and elevation of OX40L expression were not observed. By stimulating the release of IL-33, alum induced Th2 immunity via IL-5 and IL-13 production in group 2 innate lymphoid cells (ILC2s) and increased MHC class II expression in antigen-presenting cells (APCs) in the lung. Our results suggest that IL-33 secretion by epithelial cell necroptosis initiates APC- and ILC2-mediated T cell activation, which is important for the enhancement of Ag-specific IgA Ab production by alum.

## Author summary

Aluminum salts have been used as adjuvants in many vaccines. Aluminum salts induce Th2 immunity and vaccine antigen-specific antibody production aluminum salts elicit adjuvant action via cytokine production. Currently, the mechanisms underlying aluminum salt function in nasal vaccination are unknown, and elucidation of the mechanism is important for the development of particulate adjuvants. This study focused on the cytokines released from dead cells as induced by aluminum salt. This study found that aluminum adjuvant caused release of the cytokine interleukin (IL)-33 from alveolar epithelial cells by inducing necrosis. IL-33 is also crucial for antigen-specific IgA antibody production by nasal vaccination. Aluminum adjuvant also induces alveolar macrophage necrosis, which is not accompanied by IL-33 release. Aluminum salt-induced IL-33 acts as an activator for group 2 innate lymphoid cells and antigen-presenting cells in the lung. This means that by developing an adjuvant that targets the release of IL-33, it may be possible to develop a highly effective nasal vaccine. IL-33 significantly contributes to the efficacy of nasal vaccines and provides new insights into the mechanisms underlying aluminum adjuvants, showing that lung parenchymal tissue, rather than macrophages and lymphocytes, is the source of IL-33.

## Introduction

Vaccines are important tools for protecting against infectious pathogens. Various vaccines, such as those for tuberculosis, Japanese encephalitis virus, hepatitis B virus, and influenza virus, have been developed; however, the development of safer and more effective vaccines is always desirable. Currently, in addition to using pathogens such as live attenuated viruses as vaccines, inactivated pathogens, extracts, or recombinant proteins have been used as vaccine antigens (Ag). To induce sufficient antigen-specific antibody (Ab) production and cytotoxic T lymphocyte (CTL) activation, adjuvants need to be added to these subunit vaccines to compensate for the lack of immunogenicity [1].

Aluminum salts (alum) have long been used as vaccine adjuvants in humans [2, 3]. Alum is thought to exert an adjuvant effect by increasing Ag uptake [4, 5]. Alum has a high ability to induce humoral immunity, such as Ab production [2]. However, alum has a low ability to induce cell-mediated immunity, and alum can induce allergies in humans [2, 6, 7]. Subsequent studies have shown that alum exerts adjuvant effects by activating the NACHT, LRR, and PYD domain-containing protein 3 (NALP3) inflammasomes via extracellular ATP, uric acid crystals, or necrosis-released dsDNA [8–10]. Fine particulates, such as alum and silica, generally do not induce the expression of pathogen-associated molecular patterns and do not directly stimulate pattern recognition receptors such as TLRs [11, 12]. In contrast, damage-associated molecular pattern molecules (DAMPs), such as dsDNA and IL-1α, have crucial roles in immune stimulation by alum [13–17]. In particular, dsDNA stimulates immunity that induces Ag-specific IgG production when administered intraperitoneally or intramuscularly [13, 14]. IL-1α release is important for innate immune activation by alum [15–17].

The importance of IL-1β in alum-induced immunity has been widely recognized [10, 18]. In contrast, NALP3 inflammasomes and IL-1β are not required for the adjuvant effect of alum, suggesting that other cytokines are critical for eliciting adjuvanticity in alum [19]. Other members of the IL-1 family include IL-1α, IL-18, and IL-33 [20]. Among these, only IL-1α is involved in alum-induced mucosal immunity in the lungs [15]. Since the IL-1 family can be released through necrotic cell death, they are considered to be alarmins or DAMPs and activate mucosal immunity [15, 21]. IL-33 is detected in peritoneal lavage fluid after intraperitoneal administration of alum [22]. In addition, although IL-33 in the peritoneal lavage fluid can induce Th2 cytokine production, its contribution to Ag-specific Ab production is small [22]. Additionally, IL-33 has been reported to negatively regulate CTL induction after subcutaneous inoculation with alum and nanoparticles [23], suggesting that IL-33 acts as a positive regulator in humoral immunity but not in cellular immunity. Among adjuvants other than alum, 2-hydroxypropyl-β-cyclodextrin (HP-β-CD) exhibits DAMP-mediated adjuvant activity [24, 25]. Interestingly, HP-β-CD contributes to adjuvant action via dsDNA release after subcutaneous inoculation [24], whereas IL-33 is released in the lungs after nasal inoculation [25]. IL-33 has been known to induce Th2 immunity via the ST2 receptor [26, 27]; however, its mechanisms of adjuvant activities, such as identifying IL-33 target cells, as typified by APCs, have not been investigated. In addition, whether such DAMPs are involved in the adjuvant effects of nasally administered alum combined vaccines has not been investigated.

In this study, we investigated the adjuvant effects of alum in nasal administration. To elucidate its mechanisms, the Ag-specific IgA Ab production-inducing ability of alum was evaluated, and the secreted DAMPs and their target cells involved in Ag-specific IgA Ab production were analyzed.

## Results

### Nasally administered alum induces IL-33-dependent Ag-specific IgA Ab production in the lung

**Fig 1:**
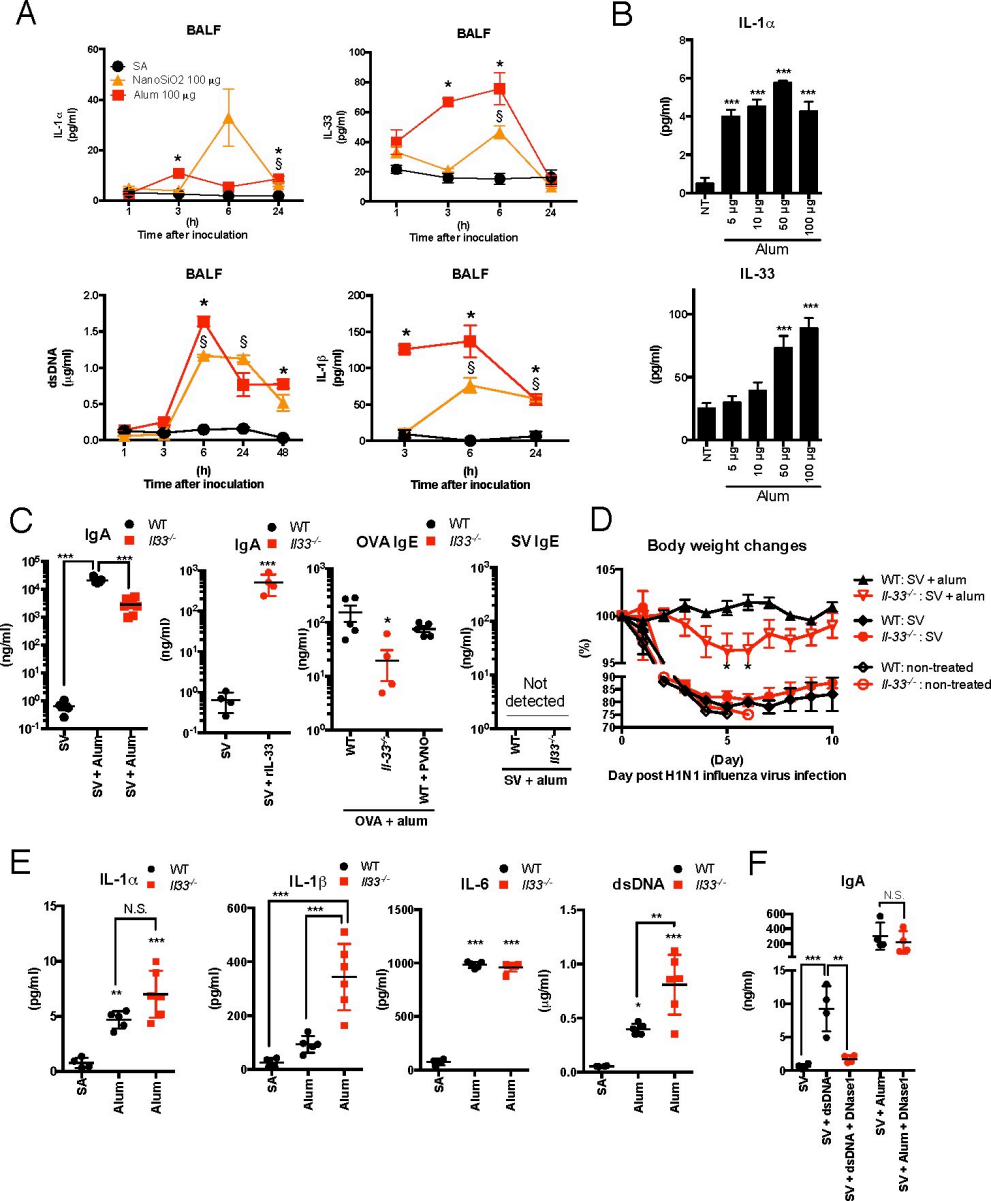
Alum has strong adjuvant effects in nasal influenza vaccines, and IL-33 has a significant influence on the induction of Ag-specific IgA Ab production by alum. (**A**) WT and IL-33-deficient (*Il33*^*-/-*^) mice were intranasally administered SA, 100 μg of alum, or 100 μg of NanoSiO_2_. At 1, 3, 6, and 24 h after administration, the mice were euthanized, and BALF was collected. The concentrations of DAMPs in BALF were determined using ELISA. Quantification of dsDNA was performed using the Quant-iT Picogreen dsDNA Assay Kit. (**B**) For dosing amount-dependent responses, 5, 10, 50, or 100 μg of alum was intranasally administered. BALF was collected 6 h and 24 h after alum intranasal administration to analyze IL-33 and IL-1α levels using ELISA. Nontreated (NT) mice were used as controls. (**C**) BALF Ag-specific IgA Ab and serum IgE Ab concentration were determined using ELISA 14 days after the second immunization with SV, SV combined with alum, SV combined with rIL-33, OVA, or OVA combined with PVNO (*n* = 4–6 per group). (**D**) Nonimmunized, SV-immunized, or SV plus alum-immunized WT or *Il33*^*-/-*^ mice were challenged with a 10 LD50 dose of A/Osaka/129/2009 [A(H1N1)pdm09] influenza virus 14 days after the second alum-adjuvanted SV immunization. Their body weight changes and survival were monitored (*n* = 4–10 per group). (**E**) BALF was collected 6 h after SA or alum intranasal administration. IL-1α, IL-1β, and IL-6 concentrations in BALF were determined using ELISA, and quantification of dsDNA was performed using the Quant-iT Picogreen dsDNA Assay Kit (*n* = 4–6 per group). (**F**) The mice were immunized with SV alone, SV with 2 μg dsDNA, or SV with alum. In some groups, 600 IU of RNase-free recombinant DNase I was intranasally administered 3 h before and 18 h after each immunization. Another group was similarly administered PBS as a control for DNase I treatment. At 14 days after immunizations, the mice were euthanized, and BALF was collected to determine Ag-specific IgA Ab concentrations using ELISA (*n* = 4 per group). The data are from two (A, B, C, E, and F) or three (D) independently performed experiments and are presented as the mean (± SEM). Each dot indicates the result of an individual animal (A, C, and D). For more than three groups, significance was assessed using one-way ANOVA and Dunnett’s multiple-comparison test to determine differences between SA and other groups. The student’s *t*-test was used to compare two groups. *p < 0.05 and ^*$*^*p* < 0.05 in the alum and NanoSiO_2_ groups compared with the SA group, respectively. *p < 0.05 and ***p < 0.001 compared with the SA group (A and C), or WT group (B). **p < 0.01 and ***p < 0.001. N.S. means not significant.

Fine particles, such as silica and alum, enhance innate immunity via DAMP release [13–17]. In this study, we found that alum and silicon dioxide nanopowder (NanoSiO_2_) are strong drivers of DAMPs in the lungs following intranasal administration (Fig 1A). Mice were intranasally administered saline (SA), alum, or NanoSiO_2_, and the DAMP concentrations in the bronchoalveolar lavage fluid (BALF) were analyzed. Alum-treated mice showed significant increases in IL-1α, IL-33, and dsDNA levels within 24 h after administration compared with the SA-administered group (Fig 1A). NanoSiO_2_-administered mice also showed significant increases in IL-1α, IL-33, and dsDNA within 24 h after administration; however, their IL-33 levels were lower than levels in the alum-administered group. IL-1β levels in the alum- or NanoSiO_2_-administered group are known to be induced by fine particle inhalation [9, 10, 18] and were also elevated within 24 h after administration (Fig 1A). Although the involvement of IL-1α and IL-1β in fine particle-induced mucosal immune responses is known [15, 21, 28–30], the role of IL-33 and dsDNA in mucosal immune responses to adjuvant activities of fine particles has not been elucidated. Dose-dependent increases in IL-33 levels were observed (Fig 1B). The concentration of IL-1α was increased by alum administration, but the effect was not dose-dependent (Fig 1B). These results indicate that alum strongly stimulates DAMP secretion in the lungs after nasal administration.

To examine the activity of alum as a mucosal vaccine adjuvant, BALB/c mice were immunized intranasally with a split influenza vaccine (SV) with or without alum. The Ag-specific IgA Ab levels in BALF were determined using ELISA. Fourteen days after the final vaccination, Ag-specific IgA Ab levels were strongly increased by alum addition compared with SV alone (Fig 1C). Furthermore, *Il33*^*-/-*^ mice showed less elevation of Ag-specific IgA Ab levels compared with those observed in wild type (WT) mice (Fig 1C). The addition of recombinant mouse IL-33 protein to the SV increased Ag-specific IgA Ab levels (Fig 1C), suggesting that IL-33 has mucosal adjuvant effects in the SV. We then assessed the OVA-specific IgE Ab response induced by OVA immunization with or without alum. Alum is known to induce high IgE Ab production in mice via OVA inhalation and is commonly used in allergy models in mice [31]. As expected, alum induced OVA-specific IgE Ab production in the serum (Fig 1C). When *Il33*^*-/-*^ mice were intranasally immunized with alum-containing OVA, OVA-specific IgE Ab levels were markedly reduced compared with those in WT mice (Fig 1C). However, when SV was used as Ag, Ag-specific IgE Ab was not detected in the serum or BALF (Fig 1C), suggesting that alum can elicit adjuvanticity without IgE Ab production in the influenza vaccine. Influenza virus infection experiments were conducted to verify the direct involvement of IL-33 in the adjuvant effects of alum. In the nonimmunized or SV-immunized study, no marked differences in influenza virus infection-induced body weight loss were observed between WT and *Il-33*^*-/-*^ mice (Fig 1D). This suggests that susceptibility to influenza virus infection and efficiency of protection in SV alone were not different between WT and *Il-33*^*-/-*^ mice (Fig 1D). In the alum containing influenza vaccine inoculation study, no marked body weight loss was observed in WT mice after infection (Fig 1D). However, *Il33*^*-/-*^ mice showed significant body weight loss compared with WT mice (Fig 1D). Taken together, these results suggest that IL-33 is a key factor in the mucosal adjuvant effects of alum.

To examine whether other DAMP and cytokine levels were affected by IL-33 defects in *Il33*^*-/-*^ mice after intranasal administration of alum, IL-1α, IL-1β, dsDNA, and IL-6 levels in BALF were analyzed 6 h after administration. No reduced levels of any DAMPs or cytokines were observed in *Il33*^*-/-*^ mice compared with WT mice 6 h after alum intranasal administration (Fig 1E). Among the markers studied, IL-1β and dsDNA increased significantly in *Il33*^*-/-*^ mice compared with WT mice (Fig 1E). These results indicate that IL-33 deficiency in *Il33*^*-/-*^ mice does not result in a decrease in DAMP secretion after alum administration. This suggests that the mechanisms underlying the decrease in Ag-specific IgA Ab production in *Il33*^*-/-*^ mice were not primarily mediated by decreases in IL-1α, IL-1β, dsDNA, and IL-6 secretion. dsDNA has been reported to have mucosal adjuvant effects in mice [29]. To investigate the adjuvant effects of dsDNA derived from alum intranasal administration, the immunized mice were treated with recombinant DNase I. The dose of dsDNA was set to 2 μg/mouse because the dsDNA concentration in the BALF of alum-administered mice was approximately 0.5–1 μg/mL (Fig 1E), and the volume of PBS used for BALF was 1.0 mL. Thus, we believe that 2 μg/mouse is sufficient to assess the effects of adjuvanticity in dsDNA. When dsDNA was combined with SV, Ag-specific IgA Ab levels increased significantly compared with SV alone (Fig 1F). DNase I treatment diminished the elevation of Ag-specific IgA Ab levels by dsDNA combined with SV vaccination, suggesting that dsDNA has mucosal adjuvant activity in SV. However, DNase I treatment did not diminish the elevation of Ag-specific IgA Ab levels by alum combined with SV vaccination (Fig 1F). These results indicate that although alum induces dsDNA release in BALF, the released dsDNA has lower effects than IL-33 on the adjuvant activities of alum. Taken together, the results suggest that IL-33 appears to be a key initiator of the mucosal adjuvant effects of alum.

### Nasally administered alum induces IL-1α release via alveolar macrophage death but not IL-33 release in the lung

**Fig 2:**
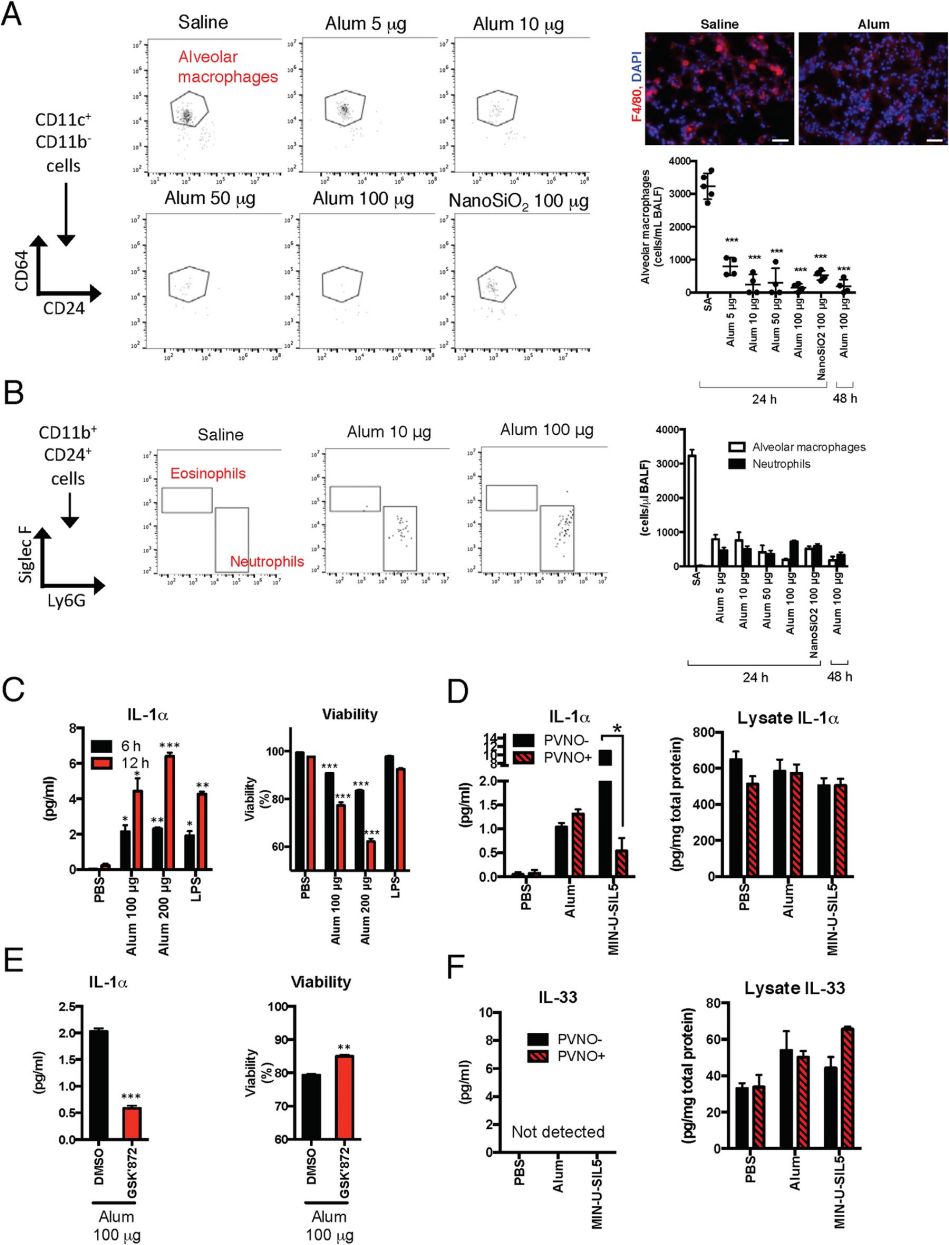
Alum induces necroptosis in freshly isolated alveolar macrophages accompanied by IL-1α secretion but not IL-33. (**A**) Mice were intranasally administered SA or 5, 10, 50, or 100 μg of alum or 100 μg of NanoSiO_2_. At 24 h or 48 h after administration, the mice were euthanized, and the lungs were collected for FACS analyses. Alveolar macrophages in the lung were gated as live (propidium iodide negative) CD11c^+^CD11b^+^CD64^+^CD24^+^. The alveolar macrophage number per mL of BALF was calculated. Freshly frozen sections of lung tissue were prepared at 24 h after intranasal administration of SA or alum and stained with anti-F4/80 Abs and DAPI. **(B)** Neutrophils and eosinophils in the lung were gated as live CD11b^+^CD24^+^Ly6G^+^SiglecF^-^ and CD11b^+^CD24^+^Ly6G^-^SiglecF^+^ cells, respectively. Gating strategies are shown in S1 Fig. Alveolar macrophage and neutrophil numbers per mL of BALF were calculated. (**C**) Freshly isolated alveolar macrophages were prepared from WT mice. Alveolar macrophages were stimulated with PBS, alum, MIN-U-SIL5, or LPS for 6 h or 12 h (only C). In some experiments, the lysosome stabilizer PVNO was cotreated with PBS, alum or MIN-U-SIL5. After stimulation, a cell-free medium was collected to determine the cell viability with the Cytotoxicity LDH Assay Kit-WST, and IL-1α and IL-33 concentrations were determined using ELISA. Cells were collected to prepare lysates for measuring IL-1α and IL-33 concentrations using ELISA and total protein concentrations with a bicinchoninic acid assay. Time- or concentration-dependent changes in IL-1α secretion and reduced cell viability. (**D**) Effects of PVNO on alum- or MIN-U-SIL5-induced IL-1α secretion. (**E**) The RIP3K inhibitor GSK’872 was treated with alum to assess the involvement of necroptosis. DMSO was added as a vehicle control. (**F**) IL-33 concentration in the medium and cell lysate after PBS, alum, or MIN-U-SIL5 stimulation with or without PVNO. The data are from three independently performed experiments, and the error bars are presented as the mean (± SEM) of three independently performed experiments. Each dot indicates the result of an individual animal (A). For more than three groups, significance was assessed using one-way ANOVA and Dunnett’s multiple-comparison test to determine differences between SA and other groups. The student’s *t*-test was used to compare two groups. *p < 0.05, **p < 0.01 and ***p < 0.001 compared with the SA group.

Programmed necrosis, also known as necroptosis, is one of several mechanisms of DAMP release [11, 20]. Previous studies have reported that intratracheal administration of alum induces alveolar macrophage necroptosis, which promotes the release of IL-1α [15]. To verify that alveolar macrophage reduction was induced during alum nasal inoculation, we analyzed the number of alveolar macrophages in BALF after alum nasal inoculation. As expected, alveolar macrophages were significantly reduced in a dose-dependent manner (Fig 2A). Total neutrophil numbers in BALF were increased, but not markedly, by alum administration (Fig 2B). Eosinophils were not found in the BALF from either group (Fig 2B). These results indicated that alveolar macrophages were reduced by the intranasal administration of alum.

To clarify whether IL-33 is directly released from alveolar macrophages, experiments using primary alveolar macrophages were performed. IL-1α, which has been known to be released from alveolar macrophages [15], was released after alum stimulation and decreased cell viability (Fig 2C). LPS was used as the control. The addition of Poly-2-vinylpyridine *N*-oxide (PVNO) as a lysosomal stabilizer suppressed the release of IL-1α by silica crystals (MIN-U-SIL5 silica), but not the release of IL-1α by alum, suggesting that alum-induced IL-1α release was not caused by lysosomal disruption (Fig 2D). *In vivo* experiments also showed that PVNO treatment suppressed IL-1α release due to NanoSiO_2_, but not alum, and that IL-33 release was not affected by PVNO treatment (S2 Fig). This suggests that the mechanisms by which alum induces cell death are different from those induced by silica. Necroptosis requires protein receptor-interacting protein kinase 3 (RIP3K) and its substrate, mixed lineage kinase domain-like (MLKL), which are crucial players in this pathway [32]. Alum-induced cell death and IL-1α release were suppressed by treatment with the necroptosis inhibitor GSK’872 as a RIP3K inhibitor (Fig 2E). These results indicate that alum induces RIP3K-mediated necroptosis in alveolar macrophages. In contrast, IL-33 release from alveolar macrophages by alum stimulation was not observed (Fig 2F). Analysis of the IL-33 protein content in the cell lysate revealed that the expression level was lower than that of IL-1α (Fig 2D and 2E). These results suggest that alveolar macrophage necroptosis releases IL-1α, but not IL-33, by alum stimulation.

### Alveolar epithelial cells express the IL-33 protein, which is released by alum-induced cell death

**Fig 3:**
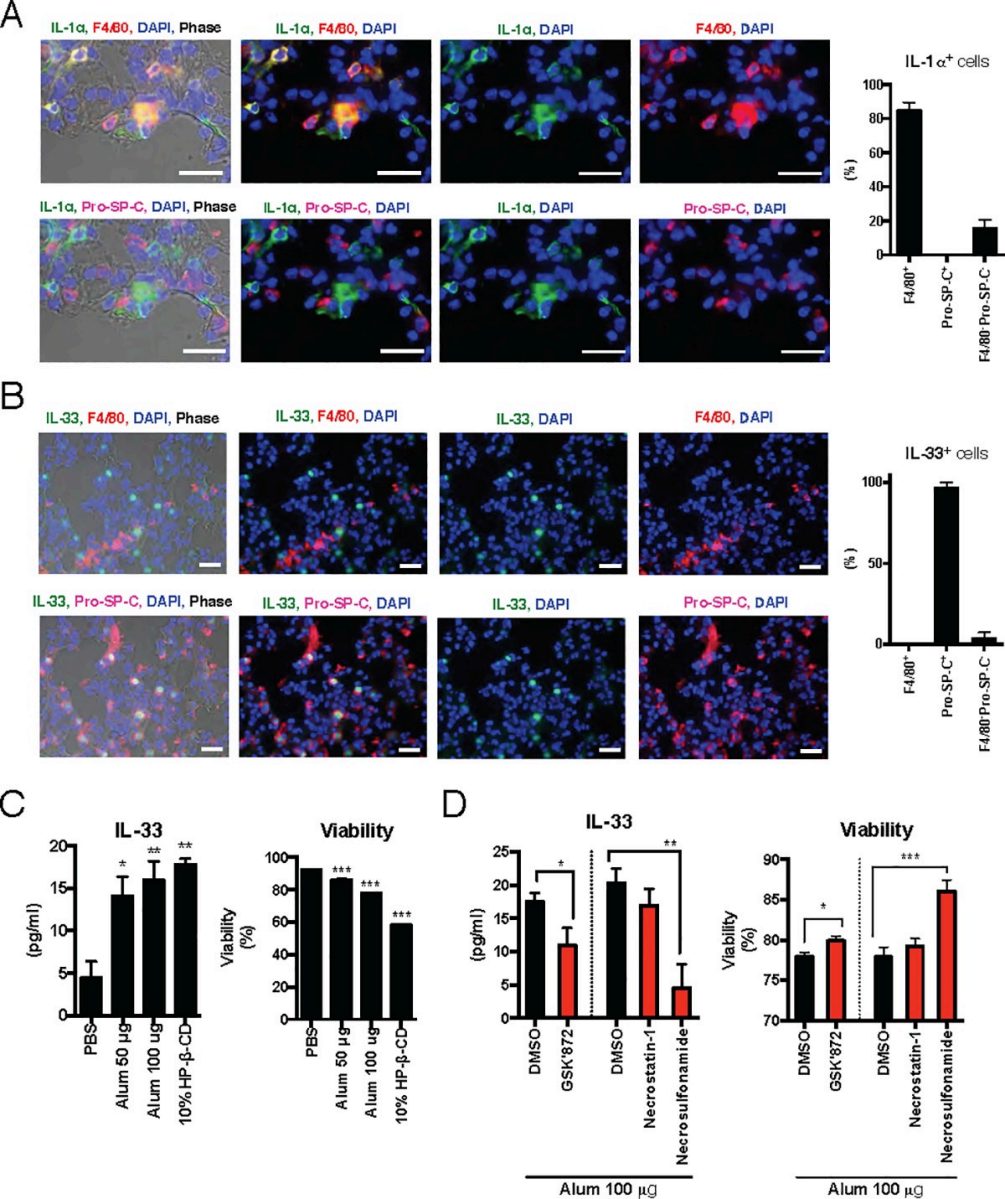
Alveolar epithelial cells lead to necroptosis-dependent IL-33 secretion. Nontreated mice were euthanized, and the lungs were collected. Fresh frozen sections were prepared and stained with anti-IL-1α Ab, anti-F4/80 Ab, anti-Pro-SP-C Ab and DAPI (**A**), or anti-IL-33 Ab, anti-F4/80 Ab, anti- Pro-SP-C Ab, and DAPI (**B**). In both **A** and **B**, the top and bottom images are from the same section, however, there are different fluorescent combinations. Images were analyzed with an Olympus BX53 fluorescence microscope. Adobe Photoshop was used to adjust the brightness and contrast (changes were applied to the entirety of all images equally). To calculate cell populations, more than 100 IL-1α^+^ or IL-33^+^ cells were counted in specimens from each animal. Among them, F4/80^+^, Pro-SP-C^+^, and F4/80^-^Pro-SP-C^-^ cells were counted, and then the ratios of each population were calculated. The data are presented as the mean ratio from three animals (±SD). **(C)** Freshly isolated alveolar epithelial cells were prepared from WT mice. The purities of the alveolar epithelial cells were determined using FACS analysis as shown in S3 Fig. The cells were stimulated with PBS, 50, or 100 μg alum or 10% HP-β-CD for 5 h. After stimulation, a cell-free medium was collected to determine the cell viability with a Cytotoxicity LDH Assay Kit-WST and the IL-33 concentration using ELISA. **(D)** To assess the involvement of necroptosis signaling, alum was coadministered with the RIP3K inhibitor GSK’872, RIP1K inhibitor necrostatin-1, or MLKL inhibitor necrosulfonamide. DMSO was added as a vehicle control. The data are from three independently performed experiments (C and D), and the error bars are presented as the mean (± SEM) of three independently performed experiments. For more than three groups, significance was assessed using one-way ANOVA and Dunnett’s multiple-comparison test to determine differences between SA and other groups. The student’s *t*-test was used to compare two groups. *p < 0.05, **p < 0.01 and ***p < 0.001 compared with the PBS group (C, D). The student’s *t*-test was used to determine differences between the DMSO- and GSK’872-treated groups, and *p < 0.05 compared with the DMSO group (E).

IL-33 is strongly expressed in parenchymal cells, such as epithelial cells, in addition to some APCs such as dendritic cells (DCs) and macrophages [27, 33]. These reports prompted us to investigate which cells release IL-33, which plays crucial roles in the adjuvanticity of nasally administered alum. To identify IL-33-expressing cells in the lung, immunofluorescence staining of the lungs was performed. F4/80 was used as an alveolar macrophage marker, and the pro-surfactant protein C (Pro-SP-C) was used as an alveolar epithelial cell marker. The results showed that many IL-1α-expressing cells also expressed F4/80 but not Pro-SP-C, suggesting that IL-1α was mainly expressed in alveolar macrophages (Fig 3A). This result supports the result shown in Fig 2C-F. In contrast, IL-33-expressing cells also expressed Pro-SP-C but not F4/80 (Fig 3B). These results show that IL-33 is strongly expressed in the alveolar epithelial cells, suggesting that alum induces necroptosis in alveolar epithelial cells and promotes IL-33 release in the lung.

To directly clarify whether alveolar epithelial cells release IL-33 by alveoli, we conducted experiments using primary alveolar epithelial cells. Mouse alveolar epithelial cells were isolated, and over 99% of the cells were CD45^-^CD31^-^, while approximately 80% of the cells were EpCAM^+^ cells, which are alveolar epithelial cells (S3 Fig). Alum induced IL-33 release by alveolar epithelial cells, accompanied by decreased cell viability (Fig 3C). HP-β-CD, which has been reported to induce IL-33 release in the lung [25], is a positive control for IL-33 release. RIP3K is phosphorylated and targets the phosphorylation of MLKL, which is critical for necroptosis [32]. In addition to RIP3K, RIP1K is also involved in the recruitment and phosphorylation of MLKL. IL-33 release and the decline in cell viability were suppressed when alum was cotreated with the RIP3K inhibitor GSK’872 (Fig 3D). However, necrostatin-1, a specific inhibitor of RIP1K, did not suppress IL-33 release or cell death (Fig 3D). Cotreatment with the MLKL inhibitor, necrosulfonamide, markedly suppressed alum-induced IL-33 release and decreased cell viability. These results show that alum promotes IL-33 release due to alveolar epithelial cell necroptosis, which is mediated by RIP3K and MLKL, but not RIP1K signaling.

### Alum can elicit Th2 immune responses via group 2 innate lymphoid cell (ILC2) activation in the lung

**Fig 4:**
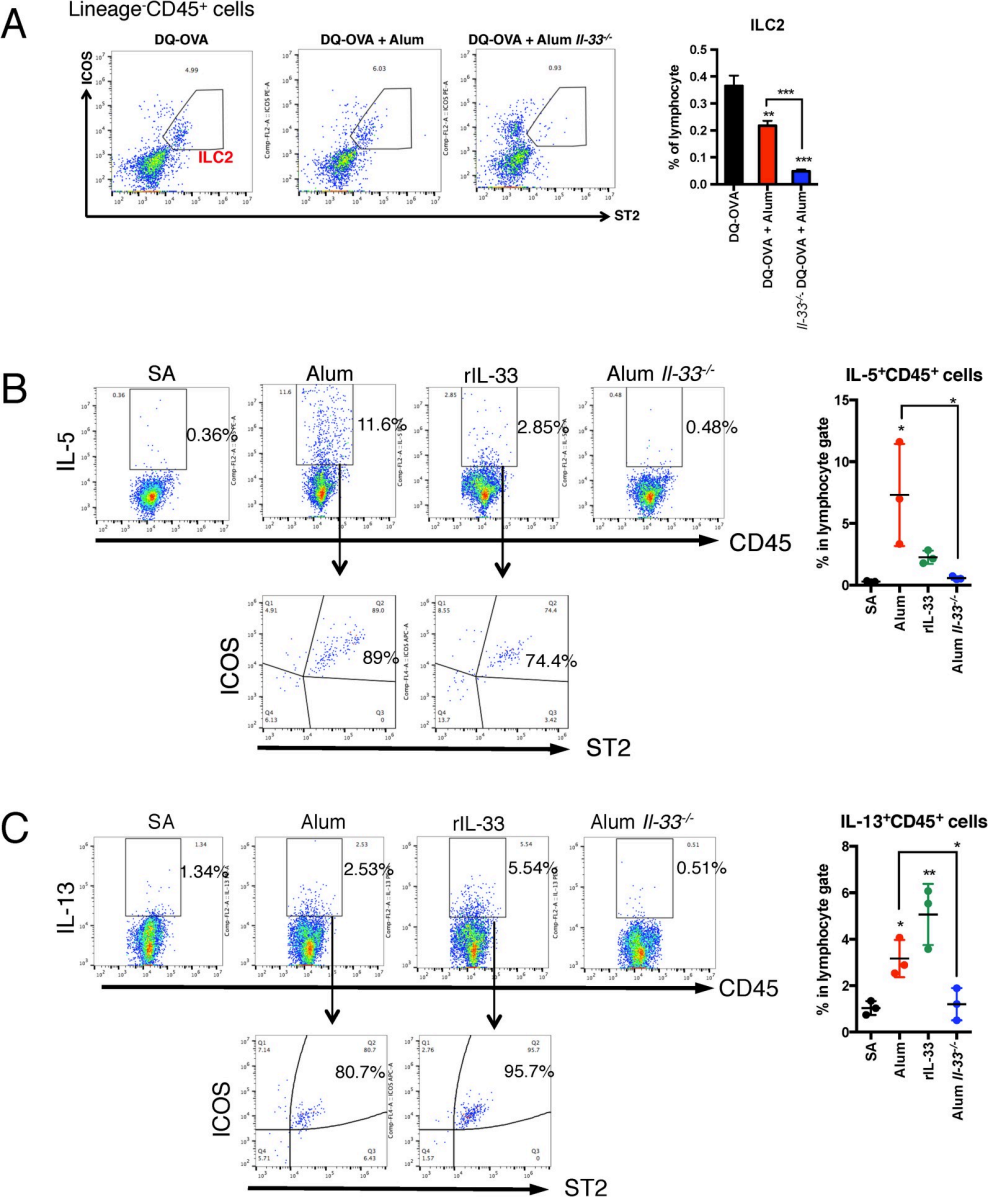
Alum increases IL-5 and IL-13 expression in lung ILC2s but does not induce ILC2 accumulation in the lung. WT or *Il33*^*-/-*^ mice were intranasally administered SA or alum. At 6 h after administration, the mice were euthanized, and the lungs were collected. ILC2s in the lung were gated as live (propidium iodide negative) CD45^+^Lineage (B220/FcεRI/CD11b/CD3ε/SiglecF)^-^ICOS^+^ST2^+^ (**A**). The ILC2 percentage of CD45^+^ cells was determined for each group (**A**). Intracellular staining with anti-IL-5 Ab (**B**) or anti-IL-13 Ab (**C**) was performed to determine their expression levels in ILC2s. The rates of CD45^+^IL-5^+^ or CD45^+^IL-13^+^ cells were calculated. Intracellular staining experiments were performed using pooled single suspensions from three mice for each group. The data are from two independently performed experiments (A–C), and the error bars are presented as the mean (± SEM) of four mice per group (A–C). Each dot indicates the result of individual experiments (B and C). For more than three groups, significance was assessed using one-way ANOVA and Dunnett’s multiple-comparison test to determine differences between SA and other groups. The student’s *t*-test was used to compare two groups. *p* * < 0.05, **p < 0.01 and ***p < 0.001 compared with the DQ-OVA group (A) or SA group (B and C).

IL-33 induces Th2-mediated adaptive immunity due to allergic inflammation in the lungs by promoting ILC2 activation and DC activation [34–36]. To verify the activation of ILC2s by nasal inoculation with alum and IL-33, the activation of ILC2s in the lungs 6 h after inoculation was analyzed using FACS. The percentage of ILC2s among total mononuclear cells in the lungs was significantly reduced in alum-administered mice (Fig 4A). In addition, *Il33*^*-/-*^ mice showed a further decrease in ILC2s compared to alum-treated WT mice (Fig 4A). Activated ILC2s are known to induce IL-5 and IL-13 expression in the lungs [37]. We analyzed the expression levels of IL-5 and IL-13 using FACS. The results showed that alum- and recombinant IL-33-administered mice had increased numbers of both IL-5- and IL-13-positive cells compared with SA-treated mice (Fig 4B and 4C). However, *Il33*^*-/-*^ mice showed no increase in IL-5- and IL-13-positive cells, despite alum administration. These results indicate that alum does not induce ILC2 accumulation in the lung; however, alum induces ILC2 activation via IL-33 secretion. In addition, IL-5-expressing cells were markedly increased by alum treatment (Fig 4B), which might be involved in the mucosal adjuvant effects of alum.

### Alum induces MHC class II expression in ST2-expressing APCs but does not induce antigen uptake in the lung

**Fig 5:**
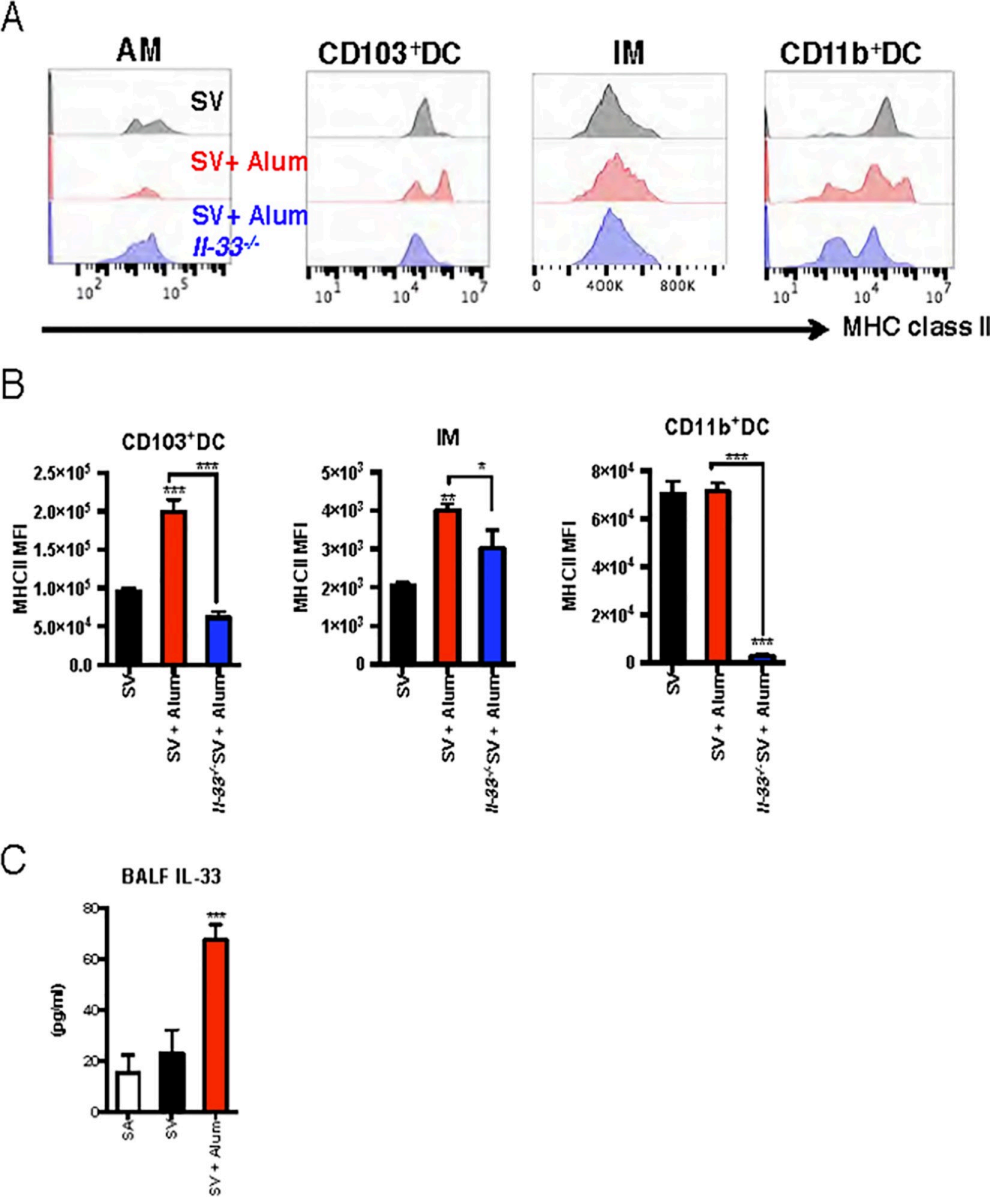
Alum increases MHC class II expression levels of APCs in an IL-33-dependent manner. WT or *Il33*^*-/-*^ mice were intranasally administered SV or SV plus alum. At 24 h after administration, the mice were euthanized, and the lungs or BALF were collected. CD103^+^ DCs, IMs, and CD11b^+^ DCs in the lung were gated as live (propidium iodide negative) CD45^+^SiglecF^-^CD103^+^CD11b^mid^CD11c^+^CD11c^+^CD24^mid^, CD45^+^SiglecF^-^CD11c^+^CD11b^+^CD24^-^, and CD45^+^SiglecF^-^CD11c^+^CD11b^+^CD24^+^, respectively. Their MHC class II expression levels were individually analyzed (**A**). The median fluorescence intensity (MFI) of MHC class II levels in each cell population was determined (**B**). The IL-33 concentrations in BALF were determined using ELISA **(C)**. The data are from three independently performed experiments (A–C), and the error bars are presented as the mean (± SEM) of three mice per group (A–C). For more than three groups, significance was assessed using one-way ANOVA and Dunnett’s multiple-comparison test to determine differences between SA and other groups. The student’s *t*-test was used to compare two groups. **p < 0.01 and ***p < 0.001 compared with the SV group (B) or SA group (C). *p < 0.05 and *** *p* < 0.001.

In addition to ILC2 activation, IL-33 promotes DC maturation and enhances Ag presentation [34]. Changes in the number of APCs in the lung and the amount of Ag uptake in APCs after intranasal administration of alum were analyzed using FACS with a gating strategy for APCs (S3 Fig). Interstitial macrophages (IMs) and CD103^+^ DCs were significantly reduced after alum administration (S3 Fig). To assess the antigen presentation ability of APCs, I-A/I-E (MHC class II) cell-surface expression levels were analyzed. CD103^+^ DCs and IM showed significant increases in MHC class II expression in the alum combined with SV-treated group compared with the SV alone-treated group, which was significantly reduced in *Il33*^*-/-*^ mice (Fig 5A and 5B). For CD11b^+^ DCs, some populations with high MHC class II expression were observed in the histogram, but when observed at the average expression level, no significant difference from SV was observed (Fig 5A). In addition, no cell population with high MHC class II expression levels was observed in *Il33*^*-/-*^ mice (Fig 5A). These results indicate that alum increases MHC class II expression levels of APCs in an IL-33-dependent manner.

**Fig 6:**
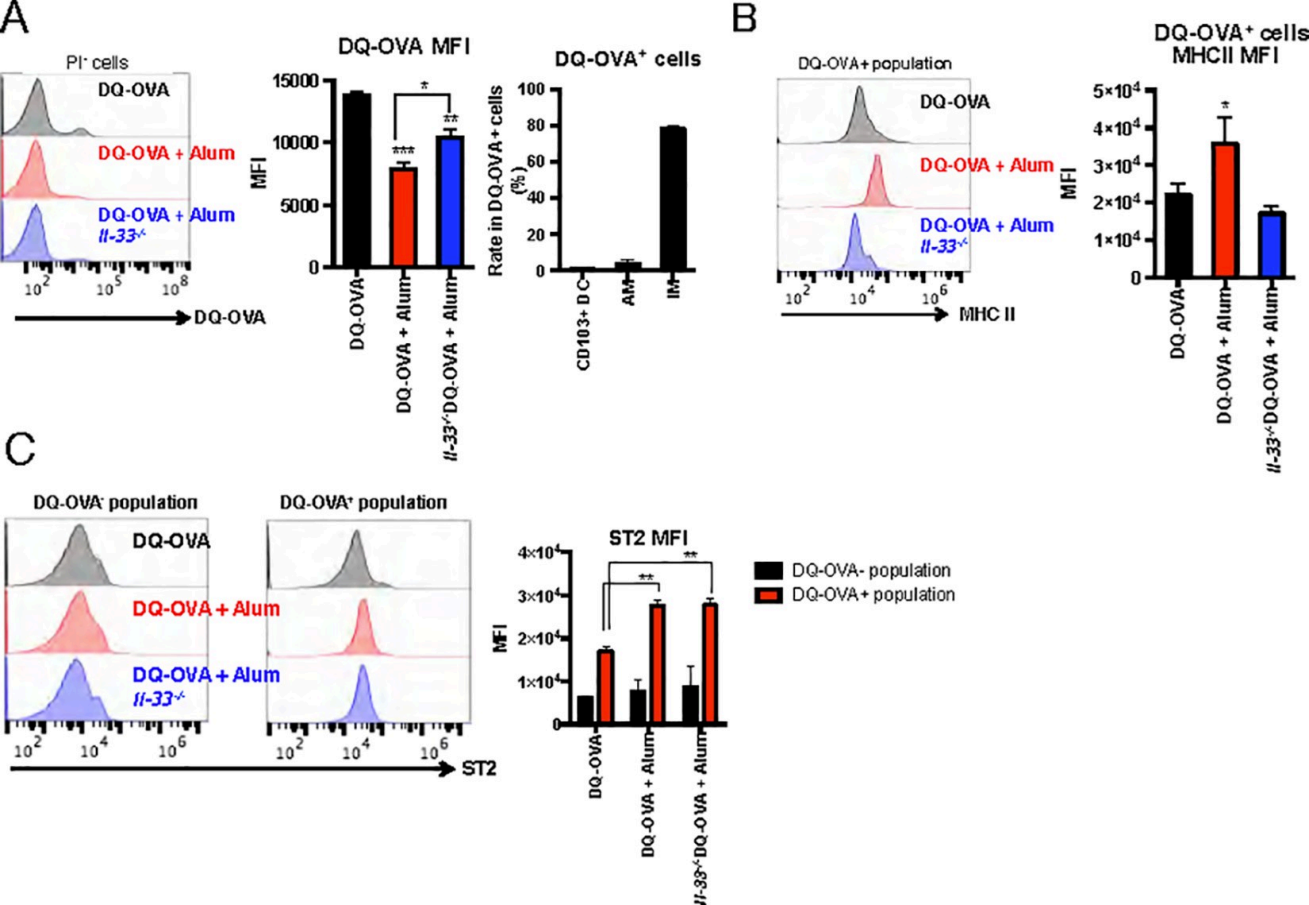
Alum increases MHC class II expression levels in OVA-uptake APCs that express ST2 in an IL-33-dependent manner. Alum also increases MHC class II expression levels of APCs in an IL-33-dependent manner. WT or *Il33*^*-/-*^ mice were intranasally administered SV or SV plus alum. At 24 h after administration, the mice were euthanized, and the lungs were collected. CD103^+^ DCs, IMs, and CD11b^+^ DCs in the lung were gated as live (propidium iodide negative) CD45^+^SiglecF^-^CD103^+^CD11b^mid^CD11c^+^CD11c^+^CD24^mid^, CD45^+^SiglecF^-^CD11c^+^CD11b^+^CD24^-^ and CD45^+^SiglecF^-^CD11c^+^CD11b^+^CD24^+^, respectively. DQ-OVA uptake levels in the total live cells and the ratio of each APC in the total DQ-OVA uptake cells (**A**), MHC class II expression levels in DQ-OVA uptake cells (**B**), and ST2 expression in both DQ-OVA uptake cells or no uptake cells were individually analyzed (**C**). The data are from three independently performed experiments (A–C), and the error bars are presented as the mean (± SEM) of three mice per group (A–C). For more than three groups, significance was assessed using one-way ANOVA and Dunnett’s multiple-comparison test to determine differences between SA and other groups. The student’s *t*-test was used to compare two groups. **p < 0.01 and ***p < 0.001 compared with the SV group (B). *p < 0.05 and ** *p* < 0.01.

To further clarify the uptake of Ag by APCs, mice were intranasally administered DQ-OVA as Ag with or without alum, and the uptake of DQ-OVA by APCs at 24 h after administration was analyzed using FACS. Although the overall uptake was very small, OVA uptake was not significantly enhanced by alum (Fig 6A), suggesting that alum does not act as an Ag uptake-inducing agent in nasal administration. In DQ-OVA-uptake cells, IM was a prominent population among the APCs (Fig 6A). To elicit adaptive immune responses, Ag presentation is crucial for APCs. MHC class II expression in DQ-OVA-uptake cells was significantly increased in alum-adjuvanted SV-administered mice compared with those administered with SV alone (Fig 6B). In addition, this observation was diminished in alum-administered *Il33*^*-/-*^ mice (Fig 6B). These results suggest that alum increases MHC class II expression levels in Ag-uptake cells via IL-33. To analyze whether the increase in MHC class II expression levels in OVA-uptake cells is mediated by the IL-33 receptor, ST2 expression levels in OVA-uptake cells were analyzed. From the result, the ST2 expression level of OVA-uptake cells was higher than that of non-OVA-uptake cells (Fig 6C). The expression level of ST2 was not markedly elevated by alum compared to DQ-OVA alone. ST2 expression levels did not change in *Il33*^*-/-*^ mice. IL-33 promotes Th2 cell activation by increasing the expression of OX40L on DCs [33, 37]. Analysis of OX40L expression levels in lung cells after alum administration did not show an increase in OX40L expression levels (S4 Fig). This suggests that increased expression of OX40L is not involved in eliciting the mucosal adjuvant effects of alum. From this, it was shown that the secretion of IL-33 rather than the elevation of ST2 expression is important for the activation of APCs by the alum.

## Discussion

Alum is an adjuvant used in many human vaccines. Since many vaccines are intended for use in healthy humans, elucidating their mechanism of action is important for clarifying their efficacy and safety. The adjuvant effects of alum have been extensively studied for many years [2, 3, 6]. Alum is a DAMPs-inducing adjuvant suggested to be involved in inducing an inoculation site-dependent characteristic immune response to alum [13–15, 23]. Most alum-containing vaccines have been inoculated subcutaneously or intramuscularly; however, fine particulates are known to activate mucosal immunity in the trachea and lungs [12]. Therefore, the present study first focused on alum-inducing DAMPs in the lungs and analyzed in detail the mechanism of action of alum as a vaccine adjuvant in nasal inoculation. This study revealed that IL-33 is released in BALF in addition to other DAMPs, such as IL-1α and dsDNA. Furthermore, IL-33 largely contributed to the induction of Ag-specific IgA Ab production by the alum-adjuvanted influenza vaccine without Ag-specific IgE Ab production. Interestingly, although neither alum nor IL-33 themselves exhibited Ag uptake-enhancing effects, alum induced IgA Ab production mainly by increasing MHC class II expression in Ag uptake APCs and promoting IL-5 and IL-13 production by activating ILC2s. These mechanisms are completely different from the mechanism of action of alum adjuvanticity reported in intramuscular, subcutaneous, and intraperitoneal administration studies [13–15, 23].

Upon intratracheal injection, a previous report showed that alum leads to necroptosis of alveolar macrophages that release IL-1α in the lungs, resulting in inducible bronchus-associated lymphoid tissue (iBALT) formation [15]. In this study and a previous study, the release of IL-1α was observed when alum was nasally inoculated (Fig 1A). In addition, IL-1β, which is released via NALP3 activation mediated by DAMPs such as dsDNA, ATP, and uric acid crystals released from injured cells [8–10], was observed in BALF (Fig 1A). In addition to IL-33, these cytokines and DAMPs are secreted after intramuscular or subcutaneous administration of alum [10, 13, 14]. Sustained activation of innate immunity promotes iBALT formation and causes toxic reactions, such as sustained IgE Ab production [15]. In contrast to IL-1α, the release of IL-33 was temporarily observed within 24 h of vaccination (Fig 1A). Furthermore, IL-33 contributed to IgE Ab production when administered with OVA (Fig 1C) but not in response to the split influenza vaccine (Fig 1C). This finding suggests that IL-33-mediated innate immune activation may be suitable as a mucosal adjuvant. Taken together, these results suggest that IL-33 is a key factor that characterizes adjuvant effects in alum nasal inoculation.

In *Il33*^*-/-*^ mice, the enhancement of Ag-specific IgA Ab production by alum was abolished without decreasing IL-1β, IL-1α, dsDNA, and IL-6 levels in BALF (Fig 1C and 1D). These results suggest that IL-33 induces adjuvant effects independent of these DAMPs and cytokine-mediated responses. In other words, the present study shows that the cytokines mainly involved in the alum-associated mechanisms of allergy and pulmonary fibrosis might be different from those involved in the induction of IgA production [9, 10, 18]. Previously, LPS-induced production of proinflammatory cytokines is increased in mice lacking the IL-33 receptor ST2 [38]. In this study, increased IL-1β production was observed in IL-33-deficient mice in response to alum administration compared with WT mice, which can be inferred to be a result of ST2-mediated regulation of TLRs and downstream signals.

A previous study demonstrated that DNA released due to host cell death indirectly induces an immune response via IL-33 [39]. Nasal inoculation with alum induced the release of dsDNA in the BALF (Fig 1A). The adjuvant effects of dsDNA were abolished by DNase I treatment (Fig 1F); however, the adjuvant effects of alum were not abolished by DNase I treatment (Fig 1F). Furthermore, no decrease in the BALF dsDNA concentration was observed in alum-treated *Il33*^*-/-*^ mice compared with alum-treated WT mice (Fig 1E). From this finding, it is predicted that unlike intraperitoneal administration and intramuscular administration [13, 14], dsDNA released due to alum nasal administration contributes little to the induction of Ag-specific IgA Ab production.

Alum leads to necroptosis in alveolar macrophages by intratracheal infusion and that IL-1α is released in the lung, resulting in the induction of iBALT formation [15]. In this study, alum induced dose-dependent decreases in alveolar macrophages in BALF (Fig 2A). As in previous reports [15], alum-stimulated cell death of primary alveolar macrophages was observed (Fig 2C). Alum concentration- and stimulation time-dependent release of IL-1α was also observed (Fig 2C). However, *in vitro* experiments in alveolar macrophages revealed that IL-33 was not released from alveolar macrophages or necroptosis (Fig 2F). Furthermore, the intracellular protein level of IL-33 was lower than that of IL-1α. Immunofluorescence staining showed that IL-33 was strongly expressed in alveolar epithelial cells in the lungs (Fig 3). Furthermore, analysis using primary alveolar epithelial cells revealed that IL-33 secretion from alveolar epithelial cells due to alum is induced by necroptosis. This suggests that the mechanism by which alum elicits characteristic immune responses depending on the inoculation site might be rooted in differences in the expression patterns of DAMPs at the inoculation site. Analyzing the immune induction mechanism based on the difference in released DAMP types depending on the inoculation site is an important issue for investigation in the future.

The mechanisms through which alum induces cell death in alveolar epithelial cells are unclear. We found that GSK’872 or necrosulfonamide significantly blocked both IL-33 secretion and cell death (Fig 3D), indicating that alum-induced cell death was RIP3K-and MLKL-dependent. However, necrostatin-1 did not suppress alum-induced IL-33 secretion and cell death in alveolar epithelial cells (Fig 3D). Therefore, we suggest that alveolar epithelial cell death is RIPK3-dependent, but RIP1K-independent necroptosis. Although RIP1K is required for TNF-induced RIP3K activation, it is not a prerequisite for TLR3-or interferon (IFN)-induced necroptosis [40]. In addition, alum-induced alveolar macrophage necroptosis is RIP3K-dependent but RIP1K-independent [15]. Taken together, these observations suggest that alum can induce necroptosis in some cells in a RIP3K-dependent but RIP1K-independent manner.

IL-33 has previously been reported to increase Th2 cytokine secretion upon intraperitoneal immunization with alum [22]. In addition, as reported, intraperitoneally injected alum-induced IL-33 has only a small effect on Ag-specific IgG1 Ab production. In this study, the Ag-specific IgA Ab production-inducing effects of alum almost completely disappeared in *Il33*^*-/-*^ mice (Fig 1C), indicating that IL-33 is crucial for the mucosal adjuvant action of alum, unlike intraperitoneal administration [22]. This may mean that IL-33 is linked to adjuvant action for intranasal routes but not for all inoculation routes. Intraperitoneal administration of alum leads to dsDNA release from dead cells and is important for Ag-specific Ab production [13], indicating that dsDNA is more important than IL-33 in eliciting adjuvant effects for intraperitoneal administration of alum. In contrast, this study showed that the role of IL-33 was more prominent than that of dsDNA in alum intranasal administration (Fig 1C and 1F). To explain the characteristic adjuvant effects depending on the inoculation site, it is necessary to analyze not only the released DAMP characteristics but also the types of cells on which DAMPs act.

IL-33 is important for eliciting mucosal adjuvant activity of HP-β-CD in the lungs [25]. The report also demonstrates the importance of IL-33 extracellular release rather than increasing its gene expression levels. Furthermore, HP-β-CD has been shown to not induce the release of IL-1α in the lungs [25]. However, the effect of IL-33 on APCs and the mechanism of induction of acquired immunity have not been verified. In this study, we analyzed the effects of IL-33 on APCs and the cell types involved in vaccination-induced acquired immunity *in vivo*. Although it has been verified in subcutaneous and intramuscular administration experiments that alum enhances Ag retention and Ag uptake by macrophages [4, 5], the present study revealed that alum or IL-33 administration does not enhance Ag uptake by intranasal administration (Fig 5A and 5B). We speculated that this phenomenon would be partially reflected in the death of alveolar macrophages due to alum administration, as indicated in Fig 2A. The ability to present Ag is important for the construction of acquired immunity. We also found that alum increased MHC class II expression in OVA-uptake cells (Fig 6B). In addition, IM accounted for most of the OVA-uptake cells, with small numbers of CD103^+^ DCs, and CD11b^+^ DCs. IL-33 enhances the Ag-presenting ability of DCs via its receptor ST2 [41, 42]. In this study, it was revealed that most of the Ag-uptake cells were ST2-expressing cells (Fig 6C), suggesting that Ag presentation might be enhanced by IL-33. In support of this hypothesis, when alum was administered to *Il33*^*-/-*^ mice, the increase in the MHC class II expression level of OVA uptake cells observed in WT mice disappeared (Fig 6B), indicating that APCs might be activated via IL-33 and ST2. However, IM accounted for OVA uptake by cells, but not DCs (Fig 6A). IL-33 polarizes alternatively activated macrophages in the lungs [38], suggesting that Ag uptake in the IM might be activated by ST2. In contrast, IL-33 also promotes Th2 cell activation through increased expression of OX40L expressed on DCs [34, 43]. Analysis of OX40L expression levels in lung cells after alum administration did not show an increase in OX40L expression levels (S4 Fig). This suggests that increased expression of OX40L is not involved in eliciting the mucosal adjuvant effects of alum.

ST2 is a receptor for IL-33 and is expressed in various cells, such as ILC2s, Th2 cells, mast cells, DCs, and macrophages [41]. ILC2s produce and secrete IL-5 and IL-13 in response to IL-33, which induces Th2 immunity [42]. IL-5 is critically involved in the enhancement of IgM production in mucosal immunity [44]. Recombinant IL-33 protein nasal inoculation increased IL-5 and IL-13 expression levels in ILC2s, and a similar phenomenon was observed with alum (Fig 4B and 4C). This study first suggests the importance of IL-33-mediated ILC2 activation as a mechanism for eliciting mucosal adjuvanticity. From the perspective of vaccine development, the regulation of acquired immunity is also important for sustained Ag-specific IgA Ab production, and elucidation of the effect of IL-33 on T cells and B cells is an important issue for future investigations.

IL-33 promotes IL-12 secretion from natural killer (NK) cells [45] and positively regulates IFN-γ production in CTLs [46] and NK cells [47]. In contrast, IL-33 acts as a negative regulator of cell-mediated immunity induced by intramuscular administration of nanoparticle adjuvants [23]. These findings suggest that IL-33 may regulate both cell-mediated and humoral immunity in a complex manner. Thus nasally administered alum-induced IL-33 may regulate not only IgA Ab production but also pulmonary CTL regulation. This is an important issue for future research.

In conclusion, this study is the first to reveal that IL-33 is important for the Ag-specific IgA Ab production-enhancing effects of alum in nasal administration. Furthermore, alum has been suggested to induce necroptosis of alveolar epithelial cells, followed by the release of IL-33, which enhances Ag-specific IgA Ab production by activating ILC2s and increasing the MHC class II level of Ag uptake by APCs. Together with findings from previous studies, it is suggested that characterizing the released DAMPs is critical to identifying the action of the adjuvant, and DAMPs may be the key factor explaining the inoculation route-specific immune response in vaccinations. These findings are important for controlling the efficacy and safety of adjuvant development.

## Methods

### Ethics statement

The animal experiments were performed according to the guidelines of the Institutional Animal Care and Use Committee of the National Institute of Infectious Diseases, Tokyo, Japan. The animal experimental protocols were reviewed and approved by the Institutional Animal Care and Use Committee of the National Institute of Infectious Diseases (approval numbers 117099 and 120100).

### Reagents

The influenza A virus (A/California/7/2009; H1N1) SV was generously provided by the Kitasato Institute (Tokyo, Japan). The following commercially available adjuvants were used: aluminum hydroxide gel (Alhydrogel, alum) obtained from InvivoGen (San Diego, California, USA). NanoSiO_2_ (particle size 10–20 nm) was obtained from Sigma-Aldrich (St. Louis, Missouri, USA). MIN-U-SIL5 silica was purchased from U.S. Silica (Berkeley Springs, West Virginia, USA). PVNO was purchased from Polysciences (Warrington, Pennsylvania, USA). Necrostatin-1 was purchased from Sigma-Aldrich. Necrosulfonamide was purchased from Cayman Chemical (Michigan, USA). GSK’872 was purchased from Abcam (Cambridge, UK). LPS (from *Escherichia coli* O55:B5, purified using phenol extraction) was purchased from Sigma-Aldrich. Low-endotoxin OVA (<1.0 endotoxin unit/mg) was purchased from FUJIFILM Wako Pure Chemical Corporation (Osaka, Japan). DQ-OVA, which is a self-quenched conjugate of OVA that exhibits bright green fluorescence upon proteolytic processing due to the released dye molecules, was purchased from Invitrogen (Carlsbad, California, USA). HP-β-CD (degree of substitution: 4.3) was generously provided by Nihon Shokuhin Kako Co., Ltd. (Tokyo, Japan). SA was purchased from Otsuka Pharmaceutical Co., Ltd. (Tokushima, Japan). RPMI 1640 medium containing 2 mM L-glutamine (Life Technologies, Carlsbad, California, USA) supplemented with 10% v/v heat-inactivated filter-sterilized FCS (GE healthcare, South Logan, Utah, USA)], 50 U/mL penicillin, and 50 μg/mL streptomycin (Life Technologies) was used to prepare complete RPMI 1640 medium for freshly isolated alveolar macrophages. Primary alveolar epithelial cells were cultured in DMEM containing 2 mM L-glutamine (Life Technologies) supplemented with 10% v/v heat-inactivated filter-sterilized FCS (GE healthcare), 50 U/mL penicillin, and 50 μg/mL streptomycin (Life Technologies). Type IV collagenase was purchased from Invitrogen, and RNase-free recombinant DNase I was purchased from Sigma-Aldrich. Carrier-free recombinant mouse IL-33 protein was purchased from BioLegend (San Diego, California, USA). The fluorescent conjugated monoclonal Abs used for FACS were as follows: fluorescein isothiocyanate (FITC)-conjugated B220 (clone RA3-6B2), FITC-conjugated FcεRI (clone MAR-1), FITC-conjugated CD3ε (clone 145-2C11), FITC-conjugated SiglecF (clone S170072), FITC or Alexa fluor700-conjugated CD11b (clone M1/70), allophycocyanin (APC)-Cy7-conjugated CD45.2 (clone 104), APC-conjugated ICOS (clone C398.4A), Brilliant Violet 421-conjugated ST2 (clone DIH9), Brilliant Violet 421-conjugated CD64 (clone X54-5/7.1), Brilliant Violet 510-conjugated Ly6G (clone 1A8), FITC or APC-conjugated SiglecF (clone S17007L), Brilliant Violet 421-conjugated F4/80 (clone BM8), Brilliant Violet 510, pacific blue or FITC-conjugated CD103 (clone 2E7), pacific blue, APC-Cy7 or Brilliant Violet 510-conjugated I-A/I-E (clone M5/114.15.2), phycoerythrin (PE)-conjugated IL-5 (clone TRFK5), and APC-conjugated OX40L (clone RM134L) were from BioLegend; FITC-conjugated CD24 (clone 30-F1), APC- or APC-eFluor 780-conjugated CD11c (clone N418), APC-eFluor 780-conjugated F4/80 (clone BM8), PE-conjugated CD45 (clone 30-F11), and PE-conjugated IL-13 (clone eBio13A) were from eBioscience/Thermo Fisher Scientific (Waltham, Massachusetts, USA); and APC-conjugated CD24 (clone M1/69), FITC-conjugated CD11c (clone N418) and PE-conjugated I-A/I-E (clone M5/114.15.2) were purchased from Invitrogen.

### Mice

Six to eight-week-old female BALB/c mice (16–22 g) were obtained from Japan SLC, Inc. (Tokyo, Japan). *Il33*^*-/-*^ mice (BALB/c background) generated in a previous study [48] were kindly provided by Prof. S. Akira (Laboratory of Host Defense, World Premier International Immunology Frontier Research Center, Osaka University, Osaka, Japan) and bred in-house. All animals were pathogen-free and maintained in individually ventilated cages. The cages were maintained at 23 ± 1°C and 50 ± 10% relative humidity under a 12 h light/12 h dark cycle. The mice were acclimated for at least 3 days before the experiments commenced. In all experiments, BALB/c and *Il33*^*-/-*^ mice were age-matched (7–12 weeks old).

### Mouse genomic DNA preparation

According to a previous study [49], mouse genomic DNA was isolated from spleens excised from female BALB/c mice using a NucleoSpin Tissue kit (Takara Bio, Tokyo, Japan) according to the manufacturer’s protocol. The prepared genomic DNA was verified using agarose gel electrophoresis. DNA quantification and purity were assessed using a NanoDrop 2000 spectrophotometer (Thermo Scientific, Waltham, Massachusetts, USA). The prepared mouse genomic DNA was used as dsDNA by mixing with SV for the immunization study.

### Immunizations

The diagrams for immunizations are shown in S5 Fig. The mice were anesthetized by intraperitoneal injection of 100 mg/kg ketamine (Ketalar; Daiichi-Sankyo Co., Ltd., Tokyo, Japan) and 10 mg/kg xylazine (Selactar; Bayer, Ltd., Tokyo, Japan), followed by intranasal immunization with SV, OVA, or DQ-OVA with or without adjuvant under anesthesia. The nasal administration volume was 15 μL for each nostril. Alum (100 μg/mouse), NanoSiO_2_ (100 μg/mouse), recombinant mouse IL-33 (500 ng/mouse), or dsDNA (2 μg/mouse) were mixed with SV or OVA. The SV, OVA, and adjuvant concentrations were adjusted by dilution with SA at a dosing volume of 30 μL/mouse. Dosing amounts of antigen protein (SV and OVA) were unified to 1 μg protein/mouse and 50 μg protein/mouse, respectively. For immunization, mice were primed on day 0 and boosted on day 14 with the same formulations. On day 28, the mice were euthanized via cervical dislocation, and BALF and blood samples were collected. BALF samples were obtained by washing the lung with 1.0 mL of PBS containing 0.1% BSA. Then, the samples were centrifuged at 1,000 × *g* for 15 min, and the supernatant was collected and stored frozen at −80°C until the measurement of Ag-specific Ab and cytokines. Sera were isolated using a Capiject (Terumo Medical Corporation, Somerset, New Jersey, USA). For *in vivo* DNase I treatment, 3 and 18 h after immunization, the mice were intranasally administered 600 IU of RNase-free recombinant DNase I as described in a previous report [49]. On day 14 after immunization, BALF and blood samples were collected and prepared as described above. For DAMP analyses, sample collection was conducted at 0, 3, 6, 24, or 48 h after primary intranasal administration.

### Cytokine and dsDNA analyses of BALF

To evaluate the levels of dsDNA, IL-33, IL-1β, IL-6, and IL-1α in BALF, the mice were injected intranasally with SA, alum (50, 10, 50, or 100 μg/mouse), or NanoSiO_2_ (100 μg/mouse). In some experiments, PVNO was intranasally coadministered with alum or NanoSiO_2_ at a dose of 100 μg/mouse, according to a previous report [15]. BALF samples were collected at 0, 3, 6, 24, and 48 h after administration. The levels of IL-1α, IL-1β, and IL-6 were measured using ELISA kits (BioLegend) in accordance with the manufacturer’s instructions. The levels of IL-33 were measured according to a previously reported method [25]. Briefly, 96-well plates were coated with 2 μg/mL purified anti-mouse IL-33 antibodies (clone Poly5165; BioLegend) in PBS overnight at 4°C. They were then washed with PBS containing 0.05% Tween-20 and incubated for 1 h with blocking buffer (PBS containing 5% FCS). After blocking, the plates were washed and incubated with diluted BALF or recombinant IL-33 overnight at 4°C. They were then washed again and incubated for 1 h with 0.5 μg/mL biotin-conjugated anti-mouse IL-33 antibody (clone Poly5165; BioLegend) in PBS with 1% BSA. The plates were washed again and incubated for 20 min with horseradish peroxidase-conjugated avidin. After a final wash, the samples were incubated with a reagent from the TMB Microwell Peroxidase Substrate System (KPL, Gaithersburg, Maryland, USA) to initiate the color reaction, in accordance with the manufacturer’s protocol. The reaction was stopped by the addition of 2 N H_2_SO_4_, and the optical density was measured at 450 nm (OD450). dsDNA in BALF was measured using the Quant-iT Picogreen dsDNA Assay Kit (Invitrogen, San Diego, California, USA) in accordance with the manufacturer’s instructions.

### FACS analysis of BALF

The BALF obtained was centrifuged at 300 × *g* for 10 min to separate the cells and fluids. For the analysis of cells in BALF, the cells were suspended in FACS buffer (5% FCS containing PBS) with anti-CD16/CD32 mAbs (Miltenyi Biotec, San Diego, California, USA) and stained with antibodies for 30 min on ice in the dark. Detailed information on the fluorescently conjugated monoclonal Abs is provided in the reagent section. The cells were washed twice with FACS buffer, resuspended in propidium iodide (BD Biosciences, San Diego, California, USA) containing FACS buffer, and then analyzed using a CytoFLEX flow cytometer (Beckman Coulter Inc., California, USA). Live cells (propidium iodide-negative cells) were also analyzed. The acquired data were analyzed using FlowJo software (TreeStar, San Carlos, California, USA). The gating strategy (S1 Fig) was created based on previous reports [15, 50].

### Measurement of antigen-specific antibody concentrations

SV Ag-specific Abs in the nasal wash, BALF, and serum samples were determined using ELISA, as previously described [51, 52]. Briefly, 96-well Falcon microtest assay plates (BD Biosciences, San Jose, California, USA) were coated with a recombinant influenza A H1N1 (A/Puerto Rico/8/34, PR8) hemagglutinin (HA) protein (Sino Biological Inc., Beijing, China) or purified A/New Caledonia/20/99; H1N1 HA protein (Kitasato Institute, Tokyo, Japan) at a 1.0 μg/mL concentration. After blocking (using 1% BSA in PBS), twofold serial dilutions of the samples or mouse anti-PR8 HA monoclonal Ab used as a standard (Sino Biological, North Wales, Pennsylvania, USA) were added and incubated overnight at 4°C. Horseradish peroxidase-labeled goat anti-mouse IgG1, IgG2a, or IgA Abs (Southern Biotechnology Associates, Birmingham, Alabama, USA) were added, and the color was allowed to develop for 15 min at room temperature in 100 μL of 1.1 mM 2,2′-azino *bis* (3-ethylbenzothiazoline-6-sulfonic acid) (EMD Biosciences, La Jolla, California, USA). The antibody concentration was calculated based on a standard curve plotted using data obtained from experiments with mouse anti-PR8 HA monoclonal antibody and recombinant PR8 protein-coated 96-well plates. In particular, the sample was serially diluted, and the antibody concentration was determined from the standard curve using a range within which the absorbance and dilution concentration were linear (*r*^*2*^ > 0.95). The concentrations of OVA-specific IgE Abs in BALF and sera were determined using the LEGEND MAX Mouse OVA Specific IgE ELISA Kit (BioLegend) in accordance with the manufacturer’s instructions.

### Influenza virus infection

The mice were primed on day 0 and boosted on day 14 with the same formulations described above. Fourteen days after the final immunization, the mice were anesthetized by intraperitoneal injection of ketamine and xylazine and then intranasally infected with A/California/7/2009 [A(H1N1)pdm09] influenza virus at a dose of 10 × LD50 in a volume of 50 μL/mouse. A diagram for immunization and virus challenge is shown in S5 Fig. All mice were monitored daily for survival and body weight loss until 10 days after infection. The humane endpoint was set at 25% body weight loss relative to the initial body weight at the time of infection.

### Primary culture cell preparation

Mouse alveolar macrophages were obtained as described previously [53]. Briefly, the lung was flushed with 0.75 mL of 37°C warmed with 2 mM EDTA and 2% FCS containing PBS 10 times. The cells were collected via centrifugation at 300 × *g* for 5 min at 4°C, and the supernatant was removed. The cells were resuspended in RPMI 1640 medium containing 10% FCS, seeded at a density of 5 × 10^5^ cells/mL, and maintained at 37°C and 5% CO_2_. Two hours after seeding, the supernatant was removed. Adherent cells were used as the freshly isolated alveolar macrophages. The purity of the alveolar macrophages was determined via FACS analysis (S1 Fig). Alveolar epithelial cell isolation was performed as described previously [54]. Briefly, the lungs were perfused with SA. Perfused lungs were lavaged once with 1.2 mL of dispase solution (BD Biosciences, 5 U/mL in 25 mM HEPES containing RPMI 1640), filled with fresh Dispase solution via a tracheal catheter, and allowed to collapse naturally. Low-melt agarose (1% w/v, 0.6 mL, 42°C) was instilled, and the lungs were immediately covered with crushed ice for 2–3 min. The lungs were removed and incubated in 2 mL of dispase solution at room temperature for 45 min on a shaker. The lungs were then teased apart and treated with 5 U/mL DNase I (Invitrogen). The digest was successively filtered through a 40 μm strainer and a 15 μm nylon mesh. Contaminating leukocytes and macrophages were depleted using MACS with biotinylated anti-CD45 (clone 104), anti-CD11c (clone N418) Abs (BioLegend), and streptavidin paramagnetic microbeads (Miltenyi Biotec). Purities were assessed using FACS analysis, and an average of > 98% was CD31^-^CD45^-^ cells. Approximately 80% of the cells were EpCAM^+^ cells, which are alveolar epithelial cells. The cells obtained were seeded into fibronectin-coated plates and cultured in DMEM supplemented with 10% FCS and antibiotics. The cells were used for assays within 48 h after seeding because the cells gradually differentiated into type 1 epithelial cells [55].

### Primary culture cell assay

For fleshly isolated alveolar macrophages, the cells were stimulated with 100 or 200 μg/mL alum, 100 μg/mL MIN-U-SIL5, or 1 μg/mL LPS for 6 or 12 h. In some experiments, an inhibitor (5 μM GSK’872 or 20 μg/mL PVNO) was added to alveolar macrophages together with alum or MIN-U-SIL5. The concentrations of particles, LPS, and inhibitors were determined according to a previous report [15]. After stimulation, the cell-free supernatants were collected and used for ELISA or cytotoxicity assays. For primary alveolar epithelial cells, the cells were stimulated with 50 or 100 μg/mL alum or 10% w/v HP-β-CD for 5 h. In some experiments, an inhibitor (5 μM GSK’872, 20 μM necrosulfonamide, or 100 μM necrostatin-1) was added to alveolar epithelial cells together with alum. After stimulation, the cell-free supernatants were collected and used for ELISA or cytotoxicity assays. ELISAs for IL-33 and IL-1α were performed as described above for the BALF cytokine analyses. Cytotoxicity was assessed using the Cytotoxicity LDH Assay Kit-WST (Dojindo, Kumamoto, Japan) in accordance with the manufacturer’s instructions.

### Immunofluorescence staining

To stain the lung tissues, the lungs were inflated from the bronchi with OCT Tissue-Tek (Sakura Finetek Japan, Tokyo, Japan) diluted 1:4 in PBS, embedded in OCT, and frozen in liquid nitrogen. The embedded tissues were stored at –80°C until analysis. Frozen sections (12 μm) of the isolated lung specimens were prepared, fixed, and permeabilized using the BD Cytofix/Cytoperm Plus Fixation/Permeabilization Kit (BD Biosciences) according to the manufacturer’s instructions [15]. The lung specimens were incubated overnight at 4°C with purified goat anti-mouse IL-33 polyclonal antibodies (Poly5165, BioLegend) or Armenian hamster anti-mouse IL-1α monoclonal antibody (clone ALF-161, BioLegend), rabbit anti-mouse Pro-SP-C polyclonal antibodies (Abcam, Massachusetts, USA, product number: ab90716), and rat anti-mouse F4/80 monoclonal antibody (clone CI:A3-1, Abcam). After washing with 5% FCS containing PBS, we stained them with Alexa Fluor488-conjugated donkey anti-goat IgG or goat anti-Armenian hamster IgG, Alexa Fluor647-conjugated donkey anti-rat IgG, and Alexa Fluor546-conjugated donkey anti-rabbit IgG (Invitrogen) for 1 h, washed with 5% FCS containing PBS, and mounted in Vector Mount with DAPI (Vector Laboratories, Burlingame, California, USA). Images were captured using an Olympus BX53 fluorescence microscope (Olympus, Tokyo, Japan). Adobe Photoshop (Adobe Systems, San Jose, California, USA) was used to adjust the brightness and contrast (changes were applied to the entire image equally). To calculate cell populations, more than 100 IL-1α^+^ or IL-33^+^ cells were counted in specimens from each animal. Among them, F4/80^+^, Pro-SP-C^+^, and F4/80^-^ProSP-C^-^ cells were counted, and the ratios of each population were calculated.

### Single-cell suspension preparation for flow cytometry analyses

For all experiments for flow cytometry analyses, immediately before collection, the lungs were perfused with PBS through the apex of the heart before tissue collection, and the lungs were inflated through the bronchi with 1 mL of digestion solution composed of 25 mM HEPES containing RPMI 1640 (Invitrogen) with 5% FCS, 10 μg/mL DNase I (Roche, Basel, Switzerland), and 1 mg/mL collagenase IV (Invitrogen). The inflated lung was placed on 2 mL of digestion solution and minced, followed by digestion for 45 min at 37°C with shaking. The reaction was stopped by placing the sample on ice with 2 mM EDTA in solution. Single-cell suspensions of lung tissue were prepared by individually mashing the tissues through a 70 μm cell strainer. The cells were pelleted at 300 *× g* for 15 min at 4°C. The supernatant was then removed. The single-cell suspensions were also RBC-lysed with ammonium chloride solution (StemCell Technologies, Vancouver, Canada) for 1 min at RT. The RBC lysates were stopped by adding a 10-fold volume of ice-cold FACS buffer (5% FCS containing PBS). The cells were pelleted at 300 *× g* for 15 min at 4°C. The cells were washed twice with ice-cold FACS buffer and suspended in FACS buffer.

### Flow cytometry analyses

Single-cell suspensions were incubated with fluorescently conjugated mAbs against mouse antigens for 30 min at 4°C. Detailed information on the antibodies is provided in the reagent section. The cells were washed twice with ice-cold FACS buffer. To exclude dead cells, the cells were suspended in propidium iodide-containing FACS buffer immediately before analysis. For intracellular staining, the cells were incubated for 4–6 h with 10 mg/mL brefeldin A (Sigma-Aldrich), 10% FCS, and 25 mM HEPES containing RPMI 1640 at 37°C. The cells were washed with FACS buffer and resuspended in FACS buffer. Fixable viability stain 510 (BD Biosciences) was used for viability staining according to the manufacturer’s instructions. After anti-CD16/CD32 mAb (Miltenyi Biotec) treatment, the cells were stained with antibodies against cell surface markers in FACS buffer for 30 min at 4°C. The cells were washed with FACS buffer before fixation and permeabilization using the BD Cytofix/Cytoperm Plus Fixation/Permeabilization Kit (BD Biosciences) according to the manufacturer’s instructions. The cells were stained with mAbs against IL-13 (eBio13A) and IL-5 (TRFK5), washed with PBS, and resuspended in FACS buffer for acquisition. Samples were run on a CytoFLEX Flow Cytometer (Beckman Coulter Inc.) and analyzed using FlowJo software (TreeStar). Gating strategies were created based on previous reports [48, 56].

### Statistical analysis

Statistical analysis was performed using GraphPad Prism software (version 6.0; GraphPad Software, La Jolla, California, USA). An unpaired Student’s *t*-test was used to compare the means of the two groups. The means of three or more groups with one variable were compared using one-way ANOVA. Where significant differences were observed, Dunnett’s multiple-comparison test was used to identify differences between the control group and other groups with an unequal number of samples. Data with values of *p* < 0.05 were considered significant. Graphs of the results are shown as the mean ± SEM of untransformed data using GraphPad Prism 6.0 software (GraphPad Software).

## Supporting information

S1 Fig Gating strategies used for FACS analysis of eosinophils and neutrophils after vaccination with primary alveolar macrophages. (A) Gating strategies used for the FACS analysis of eosinophils and neutrophils. (B) Gating strategies used for FACS analysis of primary alveolar macrophages and associated impurities. Relative to Fig 2.
(TIF)

S2 Fig Effects of lysosome stabilizer on DAMP secretion by intranasal administration of fine particles. Mice were intranasally administered 100 mg/mouse of alum or NanoSiO_2_ with or without 100 μg/mouse PVNO. At 6 or 24 h after administration, the BALF was collected to analyze the DAMPs. Concentrations of IL-1α (A) and IL-33 (B) were determined using ELISA. The data are from two independently performed experiments (A and B), and the error bars are presented as the mean (± SEM) of four mice per group (A and B). The student’s *t*-test was performed to determine the differences between the PVNO^-^ and PVNO^+^ groups. *p < 0.05. Relative to Fig 2.
(TIF)

S3 Fig Gating strategy for mouse alveolar epithelial cells and APCs, and analyses of APC population after alum intranasal administration. (B) Gating strategies used for the FACS analysis of mouse alveolar epithelial cells. Relative to Fig 3. (B) Gating strategies used for FACS analysis of alveolar macrophages (AMs), CD103^+^ DCs, interstitial macrophages, and CD11b^+^ DCs. Relative to Figs 5–6 and S3. Evaluation of APCs after intranasal administration of alum Mice were intranasally administered with SV with or without 100 μg/mouse alum. At 24 h after administration, the lungs were collected to analyze the APCs. The APC percentages of CD45^+^ cells were determined for each group. The data are from three independently performed experiments (C), and the error bars are presented as the mean (± SEM) of three mice per group (C). Significance was assessed using one-way ANOVA and Dunnett’s multiple-comparison test to determine the differences between SA and the other groups. **p < 0.01 and ***p < 0.001 compared with SV group. *p < 0.05. Relative to Fig 5.
(TIF)

S4 Fig Evaluation of OX40L expression after intranasal administration of alum. Mice were intranasally administered SV with or without 100 μg/mouse alum. At 24 h after administration, the lungs were collected to analyze OX40L expression. The OX40L MFI of the total mononuclear cells was determined for each group. The data are from three independent experiments, and error bars are presented as the mean (± SEM) of three mice per group. Significance was assessed using one-way ANOVA and Dunnett’s multiple-comparison test to determine the differences between SA and the other groups. Relative to Fig 6.
(TIF)

S5 Fig Schematic diagram of the immunization studies related to Fig 1. (TIF)

## References

[1] Reed SG, Orr MT, Fox CB. Key roles of adjuvants in modern vaccines. Nat Med. 2013;19(12):1597–608. doi: 10.1038/nm.3409 24309663

[2] Gupta RK. Aluminum compounds as vaccine adjuvants. Adv Drug Deliv Rev. 1998;32(3):155–172. doi: 10.1016/s0169-409x(98)00008-8 10837642

[3] Marrack P, McKee AS, Munks MW. Towards an understanding of the adjuvant action of aluminium. Nat Rev Immunol. 2009;9(4):287–93. doi: 10.1038/nri2510 19247370 PMC3147301

[4] Ghimire TR, Benson RA, Garside P, Brewer JM. Alum increases antigen uptake, reduces antigen degradation and sustains antigen presentation by DCs in vitro. Immunol Lett. 2012;147(1–2):55–62. doi: 10.1016/j.imlet.2012.06.002 22732235 PMC3477319

[5] Rimaniol AC, Gras G, Verdier F, Capel F, Grigoriev VB, Porcheray F, et al. Aluminum hydroxide adjuvant induces macrophage differentiation towards a specialized antigen–presenting cell type. Vaccine. 2004;22(23–24):3127–35. doi: 10.1016/j.vaccine.2004.01.061 15297065

[6] Oleszycka E, Lavelle EC. Immunomodulatory properties of the vaccine adjuvant alum. Curr Opin Immunol. 2014;28:1–5. doi: 10.1016/j.coi.2013.12.007 24463269

[7] Jensen–Jarolim E. Aluminium in Allergies and Allergen immunotherapy. World Allergy Organ J. 2015;8: 7. doi: 10.1186/s40413-015-0060-5 25780491 PMC4348159

[8] Li H, Willingham SB, Ting JP, Re F. Cutting Edge: Inflammasome activation by Alum and Alum’s adjuvant effect are mediated by NLRP3. J Immunol. 2008 Jul 1;181(1):17–21. doi: 10.4049/jimmunol.181.1.17 18566365 PMC2587213

[9] Eisenbarth SC, Colegio OR, O’Connor W, Sutterwala FS, Flavell RA. Crucial role for the Nalp3 inflammasome in the immunostimulatory properties of aluminium adjuvants. Nature. 2008;453(7198):1122–6. doi: 10.1038/nature06939 18496530 PMC4804622

[10] Franchi L, Núñez G. The Nlrp3 inflammasome is critical for aluminium hydroxide–mediated IL–1β secretion but dispensable for adjuvant activity. Eur J Immunol. 2008;38(8):2085–9. doi: 10.1002/eji.200838549 18624356 PMC2759997

[11] Zindel J, Kubes P. DAMPs, PAMPs, and LAMPs in Immunity and Sterile Inflammation. Annu Rev Pathol. 2020;15:493–518. doi: 10.1146/annurev-pathmechdis-012419-032847 31675482

[12] Kuroda E, Coban C, Ishii KJ. Particulate adjuvant and innate immunity: past achievements, present findings, and future prospects. Int Rev Immunol. 2013;32(2):209–20. doi: 10.3109/08830185.2013.773326 23570316 PMC3632271

[13] Marichal T, Ohata K, Bedoret D, Mesnil C, Sabatel C, Kobiyama K, et al. DNA released from dying host cells mediates aluminum adjuvant activity. Nat Med. 2011;17(8):996–1002. doi: 10.1038/nm.2403 21765404

[14] McKee AS, Burchill MA, Munks MW, Jin L, Kappler JW, Friedman RS, et al. Host DNA released in response to aluminum adjuvant enhances MHC CLASS II class II–mediated antigen presentation and. prolongs CD4 T–cell interactions with dendritic cells. Proc Natl Acad Sci U S A. 2013;110(12):E1122–31. doi: 10.1073/pnas.1300392110 23447566 PMC3607057

[15] Kuroda E, Ozasa K, Temizoz B, Ohata K, Koo CX, Kanuma T, et al. Inhaled Fine Particles Induce Alveolar Macrophage Death and Interleukin–1α Release to Promote Inducible Bronchus–Associated Lymphoid Tissue Formation. Immunity. 2016;45(6):1299–1310. doi: 10.1016/j.immuni.2016.11.010 28002730

[16] Oleszycka E, Moran HB, Tynan GA, Hearnden CH, Coutts G, Campbell M, et al. IL–1α and inflammasome–independent IL–1β promote neutrophil infiltration following alum vaccination. FEBS J. 2016;283(1):9–24. doi: 10.1111/febs.13546 26536497

[17] Rabolli V, Badissi AA, Devosse R, Uwambayinema F, Yakoub Y, Palmai-Pallag M, et al. The alarmin IL–1α is a master cytokine in acute lung inflammation induced by silica micro–and nanoparticles. Part Fibre Toxicol. 2014;11:69. doi: 10.1186/s12989-014-0069-x 25497724 PMC4279463

[18] Li H, Nookala S, Re F. Aluminum hydroxide adjuvants activate caspase–1 and induce IL–1beta and IL–18 release. J Immunol. 2007;178(8):5271–6. doi: 10.4049/jimmunol.178.8.5271 17404311

[19] McKee AS, Munks MW, MacLeod MK, Fleenor CJ, Van Rooijen N, Kappler JW, et al. Alum induces innate immune responses through macrophage and mast cell sensors, but these sensors are not required for alum to act as an adjuvant for specific immunity. J Immunol. 2009;183(7):4403–14. doi: 10.4049/jimmunol.0900164 19734227 PMC2912728

[20] Martin SJ. Cell death and inflammation: the case for IL–1 family cytokines as the canonical DAMPs of the immune system. FEBS J. 2016;283(14):2599–615. doi: 10.1111/febs.13775 27273805

[21] Johnson–Weaver BT, Abraham SN, Staats HF. Chapter 10 –Innate Immunity–Based Mucosal Modulators and Adjuvants. In Mucosal Vaccines, 2^nd^ ed. Kiyono H., and Pascual H. W., eds. Academic Press, San Diego, CA. 2020;167–183. doi: 10.3389/fimmu.2020.599637

[22] Rose WA 2nd, Okragly AJ, Patel CN, Benschop RJ. IL–33 released by alum is responsible for early cytokine production and has adjuvant properties. Sci Rep. 2015;5:13146. doi: 10.1038/srep13146 26272855 PMC4536651

[23] O’Grady K, Hearnden CCH, Bento D, Oleszycka E, Andersen P, Muñoz-Wolf N, et al. IL–33 Is a Negative Regulator of Vaccine–Induced Antigen–Specific Cellular Immunity. J Immunol. 2019;202(4):1145–1152. doi: 10.4049/jimmunol.1800833 30642984

[24] Onishi M, Ozasa K, Kobiyama K, Ohata K, Kitano M, Taniguchi K, et al. Hydroxypropyl–β–cyclodextrin spikes local inflammation that induces Th2 cell and T follicular helper cell responses to the coadministered antigen. J Immunol. 2015;194(6):2673–82. doi: 10.4049/jimmunol.1402027 25681338 PMC4470223

[25] Kobari S, Kusakabe T, Momota M, Shibahara T, Hayashi T, Ozasa K, et al. IL–33 Is Essential for Adjuvant Effect of Hydroxypropyl–β–Cyclodexrin on the Protective Intranasal Influenza Vaccination. Front Immunol. 2020;11:360. doi: 10.3389/fimmu.2020.00360 32210964 PMC7069475

[26] De la Fuente M, MacDonald TT, Hermoso MA. The IL–33/ST2 axis: Role in health and disease. Cytokine Growth Factor Rev. 2015 Dec;26(6):615–23. doi: 10.1016/j.cytogfr.2015.07.017 26271893

[27] Schmitz J, Owyang A, Oldham E, Song Y, Murphy E, McClanahan TK, et al. IL–33, an interleukin–1–like cytokine that signals via the IL–1 receptor–related protein ST2 and induces T helper type 2–associated cytokines. Immunity. 2005;23(5):479–90. doi: 10.1016/j.immuni.2005.09.015 16286016

[28] Lapuente D, Storcksdieck Genannt Bonsmann M, Maaske A, Stab V, Heinecke V, Watzstedt K, et al. IL–1β as mucosal vaccine adjuvant: the specific induction of tissue–resident memory T cells improves the heterosubtypic immunity against influenza A viruses. Mucosal Immunol. 2018;11(4):1265–1278. doi: 10.1038/s41385-018-0017-4 29545648

[29] Cassel SL, Eisenbarth SC, Iyer SS, Sadler JJ, Colegio OR, Tephly LA, et al. The Nalp3 inflammasome is essential for the development of silicosis. Proc Natl Acad Sci U S A. 2008;105(26):9035–40. doi: 10.1073/pnas.0803933105 18577586 PMC2449360

[30] Hornung V, Bauernfeind F, Halle A, Samstad EO, Kono H, Rock KL, et al. Silica crystals and aluminum salts activate the NALP3 inflammasome through phagosomal destabilization. Nat Immunol. 2008;9(8):847–56. doi: 10.1038/ni.1631 18604214 PMC2834784

[31] Nordvall SL, Grimmer O, Karlsson T, Björkstén B. Characterization of the mouse and rat IgE antibody responses to timothy pollen by means of crossed radioimmunoelectrophoresis. Allergy. 1982;37(4):259–64. doi: 10.1111/j.1398-9995.1982.tb01908.x 7137528

[32] Silke J, Rickard JA, Gerlic M. The diverse role of RIP kinases in necroptosis and inflammation. Nat Immunol. 2015;16(7):689–97. doi: 10.1038/ni.3206 26086143

[33] Moussion C, Ortega N, Girard JP. The IL–1–like cytokine IL–33 is constitutively expressed in the nucleus of endothelial cells and epithelial cells in vivo: a novel ’alarmin’? PLoS One. 2008 Oct 6;3(10):e3331. doi: 10.1371/journal.pone.0003331 18836528 PMC2556082

[34] Besnard AG, Togbe D, Guillou N, Erard F, Quesniaux V, Ryffel B. IL–33–activated dendritic cells are critical for allergic airway inflammation. Eur J Immunol. 2011;41(6):1675–86. doi: 10.1002/eji.201041033 21469105

[35] Rank MA, Kobayashi T, Kozaki H, Bartemes KR, Squillace DL, Kita H. IL–33–activated dendritic cells induce an atypical TH2–type response. J Allergy Clin Immunol. 2009 May;123(5):1047–54. doi: 10.1016/j.jaci.2009.02.026 19361843 PMC2711963

[36] Halim TY, Steer CA, Mathä L, Gold MJ, Martinez-Gonzalez I, McNagny KM, et al. Group 2 innate lymphoid cells are critical for the initiation of adaptive T helper 2 cell–mediated allergic lung inflammation. Immunity. 2014;40(3):425–35. doi: 10.1016/j.immuni.2014.01.011 24613091 PMC4210641

[37] Klein Wolterink RG, Kleinjan A, van Nimwegen M, Bergen I, de Bruijn M, Levani Y, et al. Pulmonary innate lymphoid cells are major producers of IL–5 and IL–13 in murine models of allergic asthma. Eur J Immunol. 2012;42(5):1106–16. doi: 10.1002/eji.201142018 22539286

[38] Brint EK, Xu D, Liu H, Dunne A, McKenzie AN, O’Neill LA, et al. ST2 is an inhibitor of interleukin 1 receptor and Toll–like receptor 4 signaling and maintains endotoxin tolerance. Nat Immunol. 2004;5(4):373–9. doi: 10.1038/ni1050 15004556

[39] Ozasa K, Temizoz B, Kusakabe T, Kobari S, Momota M, Coban C, et al. Cyclic GMP–AMP Triggers Asthma in an IL–33–Dependent Manner That Is Blocked by Amlexanox, a TBK1 Inhibitor. Front Immunol. 2019;10:2212. doi: 10.3389/fimmu.2019.02212 31616416 PMC6775192

[40] Dillon CP, Weinlich R, Rodriguez DA, Cripps JG, Quarato G, Gurung P, et al. RIPK1 blocks early postnatal lethality mediated by caspase-8 and RIPK3. Cell. 2014;157(5):1189–202. doi: 10.1016/j.cell.2014.04.018 24813850 PMC4068710

[41] Kurowska-Stolarska M, Stolarski B, Kewin P, Murphy G, Corrigan CJ, Ying S, et al. IL–33 amplifies the polarization of alternatively activated macrophages that contribute to airway inflammation. J Immunol. 2009;15;183(10):6469–77. doi: 10.4049/jimmunol.0901575 19841166

[42] Griesenauer B, Paczesny S. The ST2/IL–33 Axis in Immune Cells during Inflammatory Diseases. Front Immunol. 2017;8:475. doi: 10.3389/fimmu.2017.00475 28484466 PMC5402045

[43] de Kleer IM, Kool M, de Bruijn MJ, Willart M, van Moorleghem J, Schuijs MJ, et al. Perinatal Activation of the Interleukin–33 Pathway Promotes Type 2 Immunity in the Developing Lung. Immunity. 2016;45(6):1285–1298. doi: 10.1016/j.immuni.2016.10.031 27939673

[44] Drake LY, Iijima K, Bartemes K, Kita H. Group 2 Innate Lymphoid Cells Promote an Early Antibody Response to a Respiratory Antigen in Mice. J Immunol. 2016;197(4):1335–42. doi: 10.4049/jimmunol.1502669 27421480 PMC4976030

[45] Ochayon DE, Ali A, Alarcon PC, Krishnamurthy D, Kottyan LC, Borchers MT, et al. IL–33 promotes type 1 cytokine expression via p38 MAPK in human NK cells. J Leukoc Biol. 2020;107(4):663–671. doi: 10.1002/JLB.3A0120-379RR 32017227 PMC7229703

[46] Yang Q, Li G, Zhu Y, Liu L, Chen E, Turnquist H, et al. IL–33 synergizes with TCR and IL–12 signaling to promote the effector function of CD8+ T cells. Eur J Immunol. 2011;41(11):3351–60. doi: 10.1002/eji.201141629 21887788 PMC3332117

[47] Liang Y, Jie Z, Hou L, Yi P, Wang W, Kwota Z, et al. IL–33 promotes innate IFN–γ production and modulates dendritic cell response in LCMV–induced hepatitis in mice. Eur J Immunol. 2015;45(11):3052–63. doi: 10.1002/eji.201545696 26249267 PMC4813322

[48] Yasuda K, Muto T, Kawagoe T, Matsumoto M, Sasaki Y, Matsushita K, et al. Contribution of IL–33–activated type II innate lymphoid cells to pulmonary eosinophilia in intestinal nematode–infected mice. Proc Natl Acad Sci U S A. 2012;109(9):3451–6. doi: 10.1073/pnas.1201042109 22331917 PMC3295287

[49] Tada R, Ohshima A, Tanazawa Y, Ohmi A, Takahashi S, Kiyono H, et al. Essential Role of Host Double–Stranded DNA Released from Dying Cells by Cationic Liposomes for Mucosal Adjuvanticity. Vaccines (Basel). 2019;8(1):8. doi: 10.3390/vaccines8010008 31892192 PMC7157664

[50] Misharin AV, Morales-Nebreda L, Mutlu GM, Budinger GR, Perlman H. Flow cytometric analysis of macrophages and dendritic cell subsets in the mouse lung. Am J Respir Cell Mol Biol. 2013;49(4):503–10. doi: 10.1165/rcmb.2013-0086MA 23672262 PMC3824047

[51] Kadowaki S, Chen Z, Asanuma H, Aizawa C, Kurata T, Tamura S. Protection against influenza virus infection in mice immunized by administration of hemagglutinin–expressing DNAs with electroporation. Vaccine. 2000;18(25):2779–88. doi: 10.1016/s0264-410x(00)00087-6 10812219

[52] Tamura SI, Asanuma H, Ito Y, Hirabayashi Y, Suzuki Y, Nagamine T, et al. Superior cross–protective effect of nasal vaccination to subcutaneous inoculation with influenza hemagglutinin vaccine. Eur J Immunol. 1992;22(2):477–81. doi: 10.1002/eji.1830220228 1537382

[53] Busch CJ, Favret J, Geirsdóttir L, Molawi K, Sieweke MH. Isolation and Long–term Cultivation of Mouse Alveolar Macrophages. Bio Protoc. 2019;9(14):e3302. doi: 10.21769/BioProtoc.3302 31909091 PMC6944498

[54] Corti M, Brody AR, Harrison JH. Isolation and primary culture of murine alveolar type II cells. Am J Respir Cell Mol Biol. 1996;14(4):309–15. doi: 10.1165/ajrcmb.14.4.8600933 8600933

[55] Driscoll B, Kikuchi A, Lau AN, Lee J, Reddy R, Jesudason E, et al. Isolation and characterization of distal lung progenitor cells. Methods Mol Biol. 2012;879:109–22. doi: 10.1007/978-1-61779-815-3_7 22610556 PMC3710291

[56] Li D, Guabiraba R, Besnard AG, Komai-Koma M, Jabir MS, Zhang L, et al. IL-33 promotes ST2-dependent lung fibrosis by the induction of alternatively activated macrophages and innate lymphoid cells in mice. J Allergy Clin Immunol. 2014;134(6):1422–1432.e11. doi: 10.1016/j.jaci.2014.05.011 24985397 PMC4258609

