# Supplementary figures and images for: Nasal alum-adjuvanted vaccine promotes IL-33 release from alveolar epithelial cells that elicits IgA production via type 2 immune responses
Source: PLoS Pathog. 2021 Aug 30;17(8):e1009890. doi: 10.1371/journal.ppat.1009890 (PMC8432758; doi:10.1371/journal.ppat.1009890)

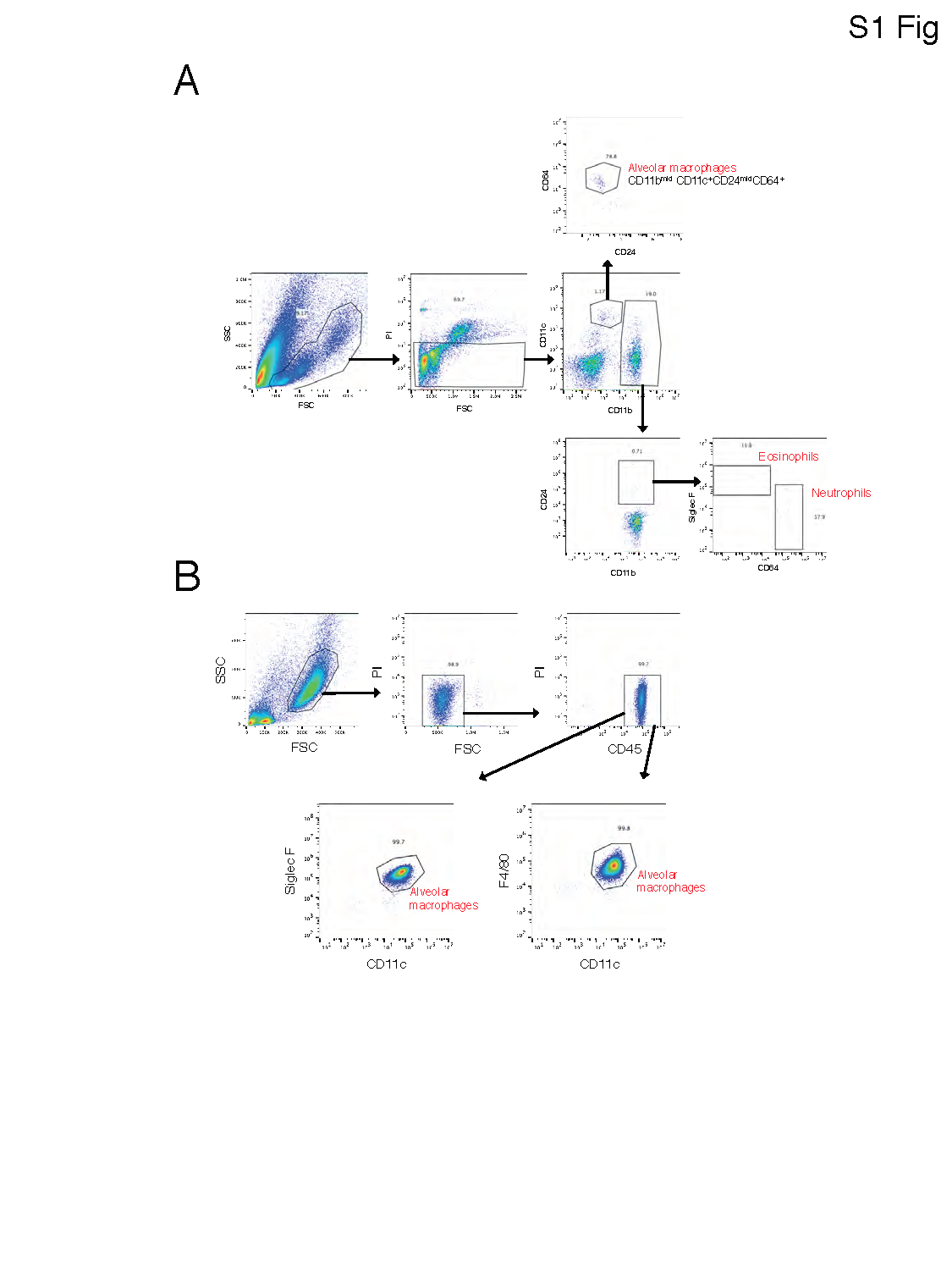

Supplement: S1 Fig — (A) Gating strategies used for the FACS analysis of eosinophils and neutrophils. (B) Gating strategies used for FACS analysis of primary alveolar macrophages and associated impurities. Relative to Fig 2. (TIF) [file ppat.1009890.s001.tif]

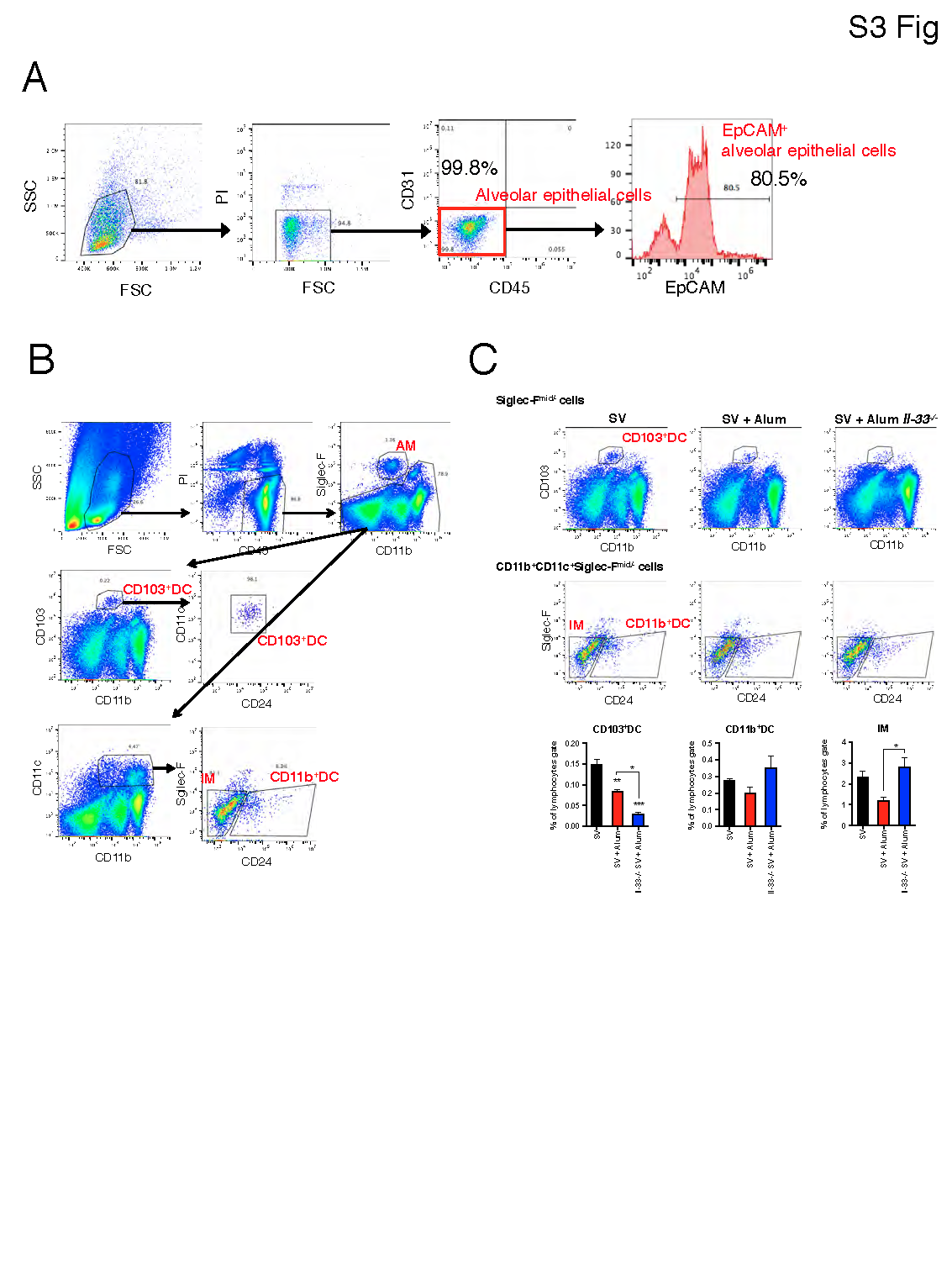

Supplement: S3 Fig — (B) Gating strategies used for the FACS analysis of mouse alveolar epithelial cells. Relative to Fig 3. (B) Gating strategies used for FACS analysis of alveolar macrophages (AMs), CD103+ DCs, interstitial macrophages, and CD11b+ DCs. Relative to Figs 5–6 and S3. Evaluation of APCs after intranasal administration of alum Mice were intranasally administered with SV with or without 100 μg/mouse alum. At 24 h after administration, the lungs were collected to analyze the APCs. The APC percentages of CD45+ cells were determined for each group. The data are from three independently performed experiments (C), and the error bars are presented as the mean (± SEM) of three mice per group (C). Significance was assessed using one-way ANOVA and Dunnett’s multiple-comparison test to determine the differences between SA and the other groups. **p < 0.01 and ***p < 0.001 compared with SV group. *p < 0.05. Relative to Fig 5. (TIF) [file ppat.1009890.s003.tif]

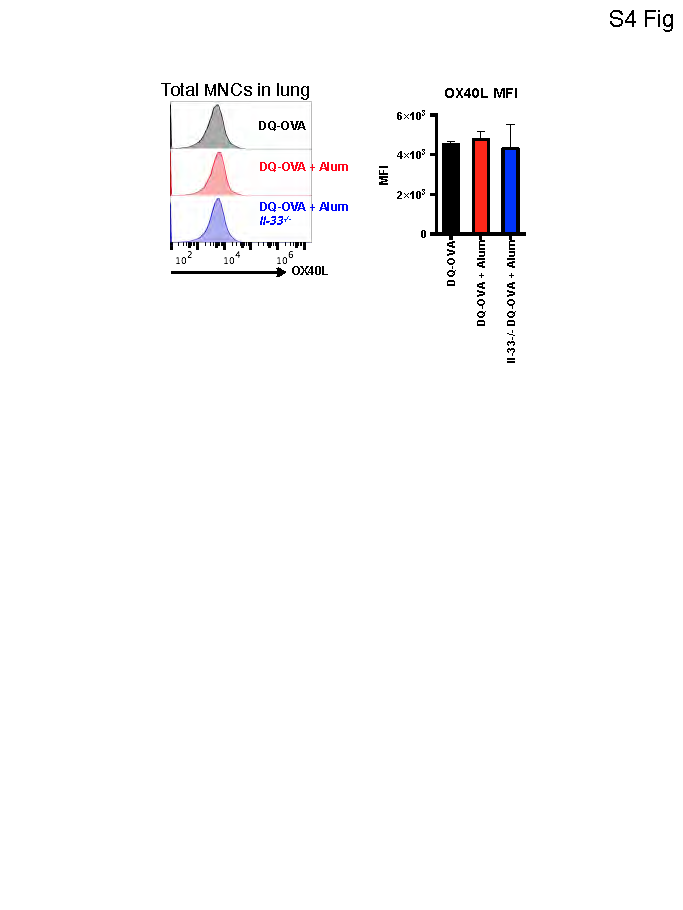

Supplement: S4 Fig — Mice were intranasally administered SV with or without 100 μg/mouse alum. At 24 h after administration, the lungs were collected to analyze OX40L expression. The OX40L MFI of the total mononuclear cells was determined for each group. The data are from three independent experiments, and error bars are presented as the mean (± SEM) of three mice per group. Significance was assessed using one-way ANOVA and Dunnett’s multiple-comparison test to determine the differences between SA and the other groups. Relative to Fig 6. (TIF) [file ppat.1009890.s004.tif]

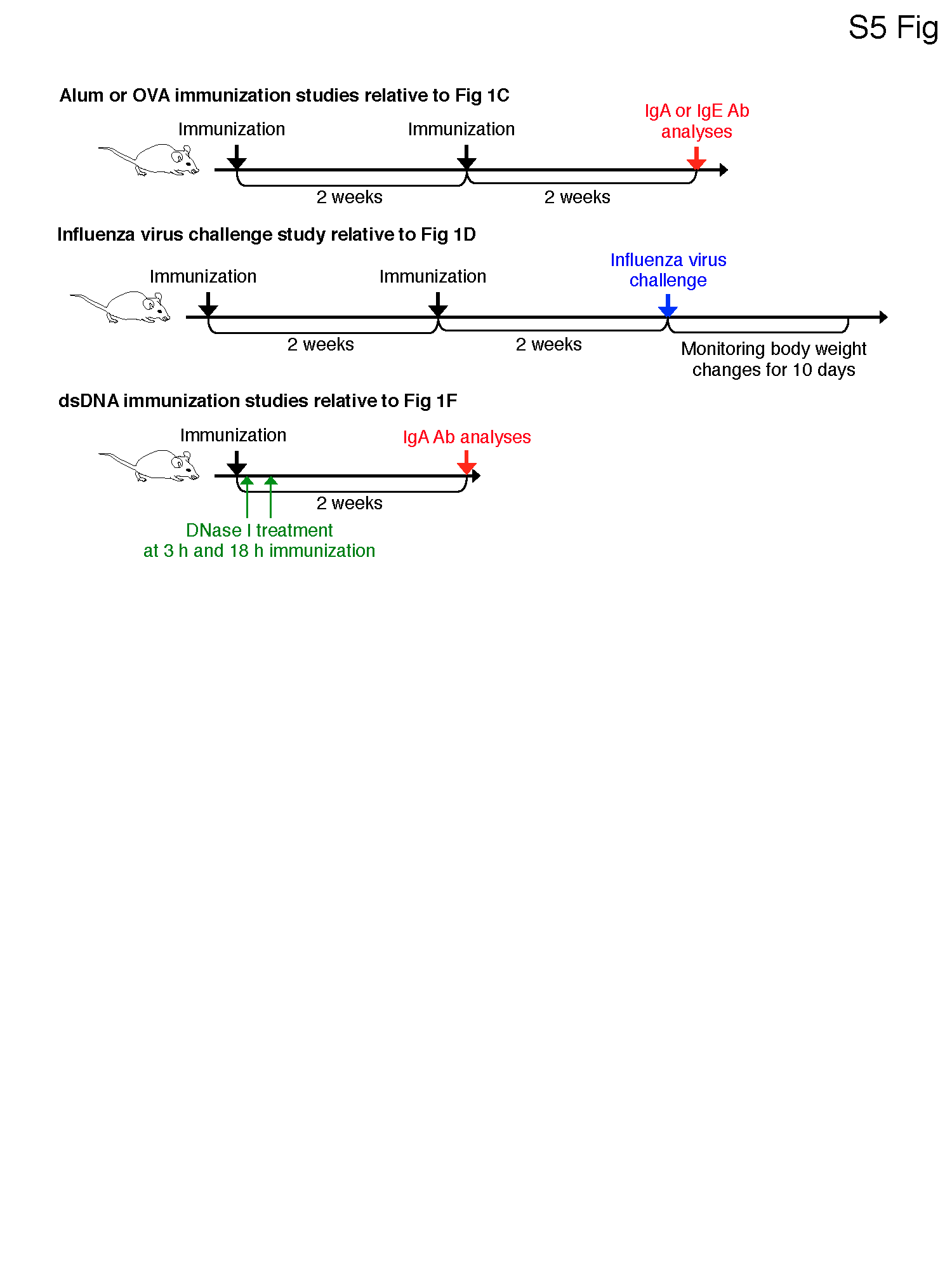

Supplement: S5 Fig — (TIF) [file ppat.1009890.s005.tif]
